# Defects of Metal Halide Perovskites in Photocatalytic Energy Conversion: Friend or Foe?

**DOI:** 10.1002/advs.202402471

**Published:** 2024-06-03

**Authors:** Chunhua Wang, Zhirun Xie, Yannan Wang, Yang Ding, Michael K. H. Leung, Yun Hau Ng

**Affiliations:** ^1^ School of Energy and Environment City University of Hong Kong 83 Tat Chee Avenue Kowloon Hong Kong SAR 999077 China; ^2^ Department of Materials Engineering KU Leuven Kasteelpark Arenberg 44 Leuven 3001 Belgium; ^3^ College of Materials and Environmental Engineering Hangzhou Dianzi University Hangzhou 310018 China

**Keywords:** charge dynamics, defect engineering, metal halide perovskites, solar‐to‐fuel conversion, surface reaction

## Abstract

Photocatalytic solar‐to‐fuel conversion over metal halide perovskites (MHPs) has recently attracted much attention, while the roles of defects in MHPs are still under debate. Specifically, the mainstream viewpoint is that the defects are detrimental to photocatalytic performance, while some recent studies show that certain types of defects contribute to photoactivity enhancement. However, a systematic summary of why it is contradictory and how the defects in MHPs affect photocatalytic performance is still lacking. In this review, the innovative roles of defects in MHP photocatalysts are highlighted. First, the origins of defects in MHPs are elaborated, followed by clarifying certain benefits of defects in photocatalysts including optical absorption, charge dynamics, and surface reaction. Afterward, the recent progress on defect‐related MHP photocatalysis, i.e., CO_2_ reduction, H_2_ generation, pollutant degradation, and organic synthesis is systematically discussed and critically appraised, putting emphasis on their beneficial effects. With defects offering peculiar sets of merits and demerits, the personal opinion on the ongoing challenges is concluded and outlining potentially promising opportunities for engineering defects on MHP photocatalysts. This critical review is anticipated to offer a better understanding of the MHP defects and spur some inspiration for designing efficient MHP photocatalysts.

## Graphical abstract

The recent progress on defect engineering of metal halide perovskites (MHPs) for photocatalysis is summarized and reexamined, focusing on discussing and critically appraising the benefits of defects, together with outlining potentially promising opportunities and directions for moving MHP‐based photocatalysis research forward.

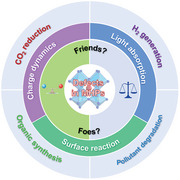

## 1 Introduction

Heterogeneous photocatalysis for solar‐to‐fuel conversion is one of the promising strategies to simultaneously address the global energy crisis and environmental pollution issues.^[^ ^1^ ^]^ For semiconductor‐based reaction systems, the photocatalytic performance of the catalysts is highly associated with energetic electrons and holes generated in semiconductors and surface reaction kinetics on photocatalysts surface. Therefore, it is well‐documented that the photocatalyst is the key factor determining the overall photocatalytic performance, and an ideal photocatalyst material is expected to embody several desirable traits:^[^ ^2^ ^]^ (i) broad light absorption, (ii) fast charge separation and transport, and (iii) abundant surface reactive sites. So far, although many photocatalysts (metal oxides, nitrides, sulfides, carbon‐based compounds, etc.) have been developed, most of them generally exhibit unsatisfactory photocatalytic activity due to poor light utilization, severe charge recombination, and sluggish surface reaction.^[^ ^3^ ^]^ Therefore, exploring novel photocatalysts to solve these issues is vital to move photocatalysis research forward.

**Figure 1:**
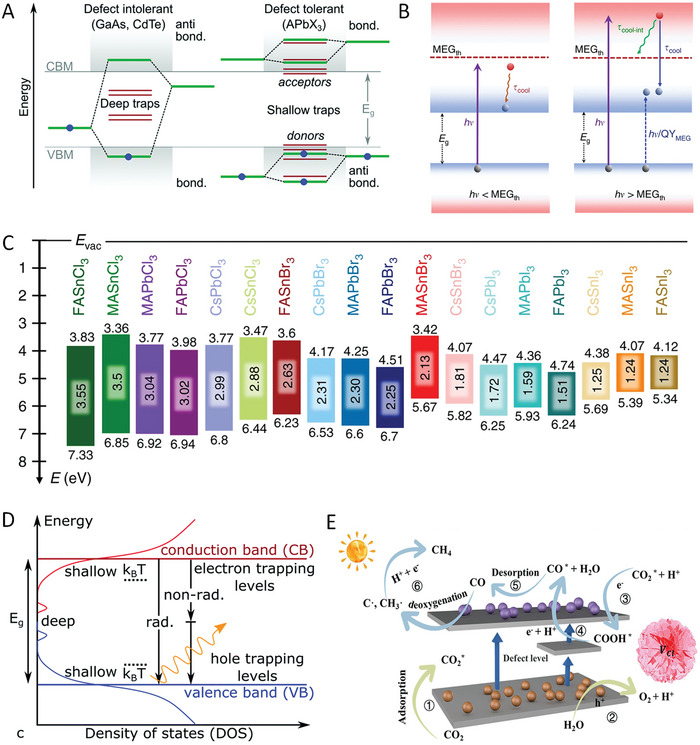
A) Comparison of the electronic band structure of traditional defect‐intolerant semiconductors (III‐V and II‐VI varieties) and defect‐tolerant MHPs. Reproduced with permission.^[^ ^4b^ ^]^ Copyright 2020, The Royal Society of Chemistry. B) Schematic illustration of hot‐carrier cooling below (left panel) and above (right panel) the multiple exciton generation (MEG) thresholds. Reproduced with permission.^[^ ^4c^ ^]^ Copyright 2018, The Authors, Published by Springer Nature. C) Schematic energy level diagram of typical MHPs. Reproduced with permission.^[^ ^5^ ^]^ Copyright 2019, The Authors, Published by Springer Nature. D) Scheme of the charge recombination caused by defects. Reproduced with permission.^[^ ^4b^ ^]^ Copyright 2020, The Royal Society of Chemistry. E) Schematic illustration of the chlorine vacancy (V_Cl_) in Cs_2_NaBiCl_6_ boosts the photocatalytic CO_2_ reduction. Reproduced with permission.^[^ ^11a^ ^]^ Copyright 2022, Wiley‐VCH.

Metal halide perovskites (MHPs) have been revolutionizing the photovoltaics field due to their fantastic optoelectronic properties,^[^ ^4^ ^]^ including high absorption coefficient (>10^5^ cm^−1^), small exciton binding energy (E_b_ < k_b_T for iodide‐based family), and long charge carrier diffusion lengths (>1 µm). Inspired by their great advances in solar cells, MHPs have recently emerged as promising photocatalysts owing to their intriguing advantages. (1) The “defect‐tolerant” characteristics (**Figure** **1**A).^[^ ^4b^ ^]^ Compared to traditional semiconductors (III‐V and II‐VI varieties), MHPs exhibit strong tolerance to defects, in which the defects generally lie near the band edges or within the bands rather than lying within the bandgap. (2) Multiple exciton generation (MEG) effects (Figure 1B).^[^ ^4c^ ^]^ Unlike conventional semiconductors which generally encounter rapid hot‐carrier cooling, MHPs can produce multiple excitons when excited by one photon with high energy, providing a route for efficient utilizing photons and multielectron redox reactions in photocatalytic processes. (3) Tunable bandgap and electronic structure (Figure 1C).^[^ ^5^ ^]^ Most traditional semiconductors have fixed bandgap positions and widths, while the bandgap of MHPs has a large region (1.2–3.6 eV) which enables the alignment of redox potential for target reactions. (4) Ease of synthesis.^[^ ^6^ ^]^ MHP photocatalysts can be easily synthesized through low‐cost solution processing, making morphology and composition engineering flexible. By virtue of these excellent properties, MHPs have been successfully used for H_2_ generation, CO_2_ reduction, organic synthesis, and pollutant degradation to date.^[^ ^7^ ^]^

Although several breakthroughs have been achieved recently, the role of defects in MHP photocatalysts is still under debate and no consensus on the exact mechanism has been reached. Following the well‐documented concept that defects in MHPs can induce large open‐circuit voltage losses in solar cells and passivating defects are very useful, it was widely considered that defects in MHP photocatalysts are detrimental to the charge recombination in its infancy (Figure 1D), and thus defect passivation has been adopted to improve the photocatalytic performance.^[^ ^8^ ^]^ Nevertheless, things may be different between solar cells and photocatalysis. On the one hand, due to the defect tolerance property, the charge carriers in defects can have similar energies compared to those in the band edge, which suggests that highly energetic carriers can potentially participate in photocatalysis.^[^ ^9^ ^]^ On the other hand, it has been reported that when precisely engineered, certain types of defects in small amounts, can improve the photocatalytic performance of some photocatalysts (TiO_2_, Bi_2_WO_6_, g‐C_3_N_4_, etc.),^[^ ^10^ ^]^ in which defects can optimize the electronic structure, tune the light absorption, facilitate the charge separation, and offer more reactive sites (Figure 1E). Notably, some recent studies have also shown that the defects in MHPs can be beneficial for photocatalytic performance.^[^ ^11^ ^]^ Theoretically, with the help of density functional theory (DFT) calculations, Pi et al. showed that the Cl vacancy in Cs_2_NaBiCl_6_ serves three functions: suppress charge recombination, enhance CO_2_ adsorption, and reduce the free energy barrier for the generation of key intermediate COOH*, which significantly boosts the CO_2_ photoreduction.^[^ ^11a^ ^]^ Similarly, He et al. revealed that the Br vacancy in Cs_2_AgBiBr_6_ tunes the local atomic arrangement and electronic structure, which facilitates charge generation and separation and thus enhances photocatalytic H_2_ generation.^[^ ^11e^ ^]^ Experimentally, Cs_2_AgBiBr_6_ with Br vacancies^[^ ^11a^ ^]^ and MAPbI_3_ with continuously distributed I defects^[^ ^11a^ ^]^ were shown to promote the adsorption/activation of CO_2_ molecules in Cs_2_AgBiBr_6_ and photogenerated charge separation in MAPbI_3_, leading to enhanced photocatalytic CO_2_ reduction and H_2_ generation, respectively. Thus, it is necessary to understand the effect of defects in MHPs and distinguish to what extent the defect could boost a photocatalytic reaction. However, a systematic summary of how the defects in MHPs affect the photoredox reactions is still lacking, making it urgent to re‐examine the role of defects in MHPs to move the MHP photocatalysis forward.

In this review, we provide a critical analysis of defects in MHP materials and highlight their roles in photocatalytic applications. First, we began by clarifying the origin of defects in MHPs, along with the classification of the defects. Second, we highlighted and discussed the potential advantages of defects in photocatalysts, including optical absorption, charge dynamics, and surface reaction kinetics. Then, we assessed the recent progress on defect engineering from two contradictory viewpoints, i.e., either passivating or exploiting defects to improve the photoactivity of MHP photocatalysis, focusing on discussing utilizing defects to boost the photocatalytic performance. Finally, we conclude by casting a personal prospect on the ongoing challenges and opportunities regarding defect‐mediated MHP photocatalytic applications. To the best of our knowledge, this is the first comprehensive review of the defects for MHP photocatalysis. We anticipate that this review will deliver a better understanding of the defects on MHPs‐based photocatalysis and offer a guideline for the research of MHP photocatalytic applications.

## 2 Origin of Defects in Metal Halide Perovskites (MHPs)

The early investigation on photocatalysts was dominated by semiconducting metal oxides, metal chalcogenides, and nitrides. In recent years, MHP materials have attracted tremendous attention owing to their skyscraping performance in solar energy harvesting and conversion.^[^ ^12^ ^]^ Especially, the state‐of‐art MHP‐based solar cell has recently reached an efficiency of 26%,^[^ ^13^ ^]^ approaching the Shockley‐Queisser limit and on par with the widely adopted silicon technology. Besides the suitable bandgap energy, another crucial reason for the rapidly climbing solar conversion efficiency of MHPs is owing to their unique defect properties.

For the perfect semiconductor crystal with uninterrupted translational symmetry, each atom periodically resides on its prescribed lattice site. The structural imperfections including the interruptions to the intact crystal lattice, or the foreign atoms introduced into the lattice sites are deemed as defects. Once the semiconductor lattices do not extend perfectly over a large distance due to the introduction of defects or impurities, some properties such as conductivity, free charge carrier mobility, and lifetime will be dramatically affected in spite of the minimal defect concentration at part per million or part per billion level.^[^ ^14^ ^]^ As an emerging light responsive material, the finer mechanism of defect‐induced effects on the performance of MHP‐based optoelectronic applications still remains obscure despite the significant endeavors have been made by the scientific community.^[^ ^15^ ^]^ The elaboration of defect engineering was proved to promote the progress and development of the well‐established semiconductors such as silicon (Si)^[^ ^16^ ^]^ and metal chalcogenide (CdTe, Cu(In,Ga)Se_2_, Cu_2_ZnSnS_4_, etc.).^[^ ^17^ ^]^ To deepen the understanding and guide the structural regulation of MHPs in photocatalysis, the physiochemical properties of defects in MHPs and their impacts will be reviewed.

### 2.1 Classification of Defects

#### 2.1.1 Point Defects

**Figure 2:**
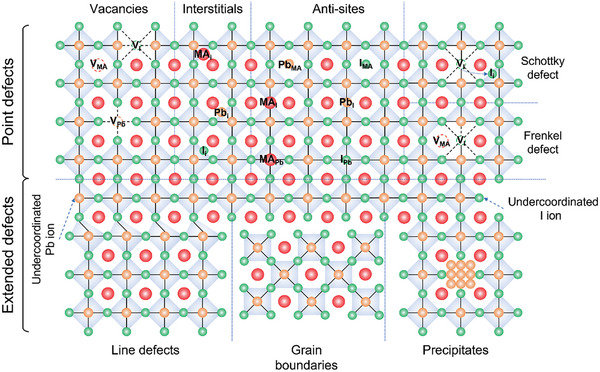
Schematic diagram of the defect types in typical MAPbI_3_ material.

Due to the soft nature, MHP absorbers synthesized from solution along with a post‐treatment process at low temperatures usually go through rapid crystallization accompanied by the expeditious generation of defects. Vacancies, interstitials, and anti‐site substitutions (**Figure** **2**) are the primary kinds of point defects in semiconductor crystals which are intrinsically formed during the crystal growth or caused by the intentional introduction of foreign species (such as dopants). The possibility of the formation of the point defects is believed to be determined by the formation energy. Yin et al. simulated 12 possible point defects in MAPbI_3_ (CH_3_NH_3_PbI_3_, methylammonium lead iodide),^[^ ^18^ ^]^ including three vacancies (V_MA_, V_Pb,_ and V_I_), three interstitial (MA_i_, Pb_i_, I_i_) and six anti‐site substitutions (MA_Pb_, MA_I_, Pb_MA_, Pb_I_, I_MA_, I_Pb_). They theoretically revealed that these point defects (I_MA_, I_Pb_, Pb_I_, Pb_i_) that contribute to the deep‐level traps have a high formation energy, suggesting a low non‐radiative recombination rate. Whereas the dominant defects with low formation energies only give rise to the shallow trap states close to conduction or valence band edges, which also induced either p‐type or n‐type doping but was less detrimental to the transport and lifetime of the charge carriers.

In addition to the individual types of point defect, the generation of neutrally charged defect pairs is also expected, such as Frenkel defect and Schottky defect. The Frenkel defect was created by a pair of vacant and interstitial sites, whereas the Schottky defect consisted of two vacancies with opposite charges. According to the density functional theory (DFT) calculation by Kim et al., the Schottky defects such as PbI_2_ and MAI, did not create trap states within the bandgap; meanwhile, vacancies derived from the Frenkel defects (such as Pb, I, and MA vacancies) serves as the unintentional doping source was demonstrated contributing to the shallow traps near the band edges.^[^ ^19^ ^]^ On the basis of theoretical and experimental results, two most abundant defects in MAPbI_3_ (V_I_ and I_i_) are more likely generated as Frenkel pairs.^[^ ^20^ ^]^

#### 2.1.2 Extended Defects

**Figure 3:**
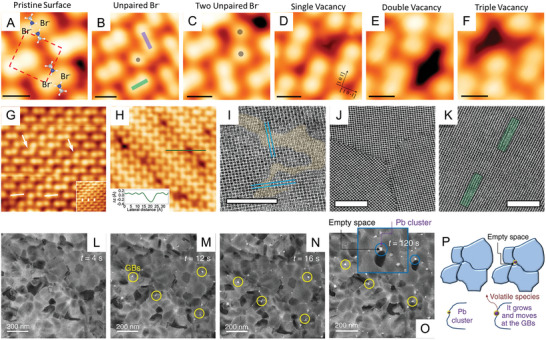
A–F) High‐resolution STM images of MAPbBr_3_. (A) Pristine surface, with MA^+^ molecules overlaid to show relative position. (B) An unpaired Br anion defect, (C) Two adjacent unpaired Br anion defects located near a vacancy. (D‐F) Single, double, and triple defects, respectively. Reproduced with permission.^[^ ^22^ ^]^ Copyright 2019, American Chemical Society. (D, H) High‐resolution STM images of MAPbBr_3_. G) Dislocations, the start of dislocation rows indicated by the white arrow, and H) defect on the surface, inset: Height profile across a defect along the green line indicated in the image. Reproduced with permission.^[^ ^21^ ^]^ Copyright 2015, American Chemical Society. I–K) Atomic‐resolution scanning transmission electron microscopy (STEM) of FAPbI_3_. (I) Native intergrowth between PbI_2_ (shaded yellow) and FAPbI_3_ formed. (J) Abrupt grain boundaries and (K) edge dislocations (green rectangle). Reproduced with permission.^[^ ^23^ ^]^ Copyright 2020, The Authors, published by American Association for the Advancement of Science. L–P) TEM images of the degradation transition of MAPbI_3_ (L‐O) Pb‐cluster at the GBs during time, yellow circles indicate Pb‐clusters at the GBs. (P) Schematic showing the Pb nano‐clusters growing at the perovskite grain boundaries and leaving empty spaces behind as shown in Figure 4O. Reproduced with permission.^[^ ^24^ ^]^ Copyright 2019, The Authors, published by Springer Nature.

The debate on whether the intrinsic point defect properties are sufficient to decipher the phenomenon in MHPs still remains ongoing. In addition to the point defect, extended defects with higher dimension orders are also observed, such as line defects, grain boundaries, and precipitates (Figure 2). The line defect is where lattice periodicity is discontinuous along a line, such as edge dislocation. The grain boundaries (GBs) are caused at the plane where the crystalline grains with different orientations meet and precipitates are clumps of impurity crystalline structure as inclusion inside the main perovskite phase, which is generally formed due to the excess or impurity of the perovskite precursor salts. Atomic‐resolution microscopes have been demonstrated as a useful tool to visualize the nano‐scale defect morphology of MHP materials. As shown in **Figure** **3**G,H, Ohmann et al. first revealed the real‐space topographic images of the dislocation and defects from an in‐situ cleaved MAPbBr_3_ single crystal with the atomic‐scale scanning tunneling microscope (STM).^[^ ^21^ ^]^ Stecker et al. also presented atomic‐scale investigation on the multiple types of defects on the surface of MAPbBr_3_ (Figure 3A–F), including the vacancy clusters (identified as MA_Br_ vacancies) and unpaired Br^−^ ions.^[^ ^22^ ^]^ With the help of atomic‐resolution transmission electron microscopy (TEM), Rothmann et al. observed varied structural imperfections in FAPbI_3_ ((HC(NH_2_)_2_)PbI_3_, formamidinium lead iodide), including the PbI_2_ impurity domains perfectly integrated in FAPbI_3_ matrix (Figure 3I), the grain boundaries with the sharp interfaces (Figure 3J) as well as the edge dislocation which is dissociated perpendicular to the glide (Figure 3K).^[^ ^23^ ^]^ The degradation of MAPbI_3_ induced by Pb‐clustering has been verified by Alberti et al. with the low electron dose TEM.^[^ ^24^ ^]^ The aggregation of Pb‐related defects and their preferential accumulation at the GBs of MAPbI_3_ were gradually observed in Figure 3L–P, leaving empty spaces at the surface of MAPbI_3_ grains. Due to their polycrystalline nature, the GBs are the most inevitable defects in the MHP thin films and are believed to determine both the efficiency and stability of MHP‐based devices.^[^ ^25^ ^]^ Although some groups have demonstrated that the GBs could facilitate the photoinduced charge separation without creating gap states^[^ ^26^ ^]^ and such statement has been evidenced by the observation from c‐AFM and KPFM,^[^ ^27^ ^]^ substantial research works defied this prediction.^[^ ^28^ ^]^ Long et al. applied the nonadiabatic molecular dynamic combined with time‐domain density functional theory and unraveled that the GBs accelerate the electron‐hole recombination rate,^[^ ^29^ ^]^ which is opposite to the first‐principle simulated results from the discovery from Yin et al. that the GBs of MAPbI_3_ are intrinsic benign.^[^ ^30^ ^]^ The shorter photoluminescence (PL) lifetime and lower PL intensity at the GBs compared to the grain interior substantiated the faster non‐radiative recombination rate at the GBs.^[^ ^28a^ ^]^ Furthermore, the GBs have been accused of being the main ion immigration channel in the polycrystalline MAPbI_3_ thin film^[^ ^31^ ^]^ as well as the more vulnerable toward degradation.^[^ ^32^ ^]^ Hence, GBs are believed to be one of the key factors for the low efficiency and instability of MHPs.

In addition to the boundary between grains, the defective interfaces of MHPs are also of great importance since the charge carrier separation and collection at the interface is dominated by the trap states at the surface of MHPs such as dangling bonds, undercoordinated ions, and chemical impurities. Theoretical simulation by DFT has revealed that the vacant terminations are thermodynamically stable compared to the PbI_2_‐rich flat terminations on the primary surfaces of MAPbI_3_.^[^ ^33^ ^]^ Wu et al. estimated the distinct difference between the photophysical properties of the surface and the bulk of MAPbBr_3_ single crystal and uncovered that the surface (≈6×10^17^ cm^−3^) has a trap density two orders of magnitude greater than that of the bulk (5.8×10^15^ cm^−3^),^[^ ^34^ ^]^ resulting in a great shortening in the charged carrier lifetime (from ≈34 to 1 ns) and carrier diffusion length (from ≈2.6–3.4 µm to ≈130–160 nm). For the case of polycrystalline films, Huang and co‐workers also demonstrated that the charge trap densities at the interface are one or two orders of magnitude greater than that of the interior films.^[^ ^35^ ^]^ Moreover, surface defects are regarded as the major reason that trigger and accelerate the degradation of perovskite materials due to their susceptibility to exoteric molecules in the environment such as H_2_O and O_2_.^[^ ^36^ ^]^ For instance, Aristidou et al. illustrated that the iodide vacancies at the defect rich surface serve as the preferential sites for photo‐induced generation of superoxide species from oxygen, which is account for the degradation of MAPbI_3_.^[^ ^37^ ^]^ Although the current research mainly shows the detrimental effects of defective surfaces in MHP‐based photovoltaic and LED devices, it should be noted that in the photo(electro)catalytic systems where more solid‐gas/liquid interfaces are involved for the primary redox reaction, the role of defect states might be different. In addition to the defect‐induced disadvantageous influences on MHPs, it is still possible that the defects might play positive roles in photocatalytic progress, which will be thoroughly discussed in part 4 of this review.

### 2.2 The Properties of Defects

#### 2.2.1 Shallow Level Defects

For many traditional semiconductor photocatalysts (such as CdSe), both their conduction band (CB) and valence band (VB) exhibit obvious bonding–antibonding states form across the bandgap, which results in the more likely generation of deep level states in the bandgap (Figure 1A).^[^ ^38^ ^]^ However, the unique crystalline and electronic structures fetch a distinctive story for the MHPs.

**Figure 4:**
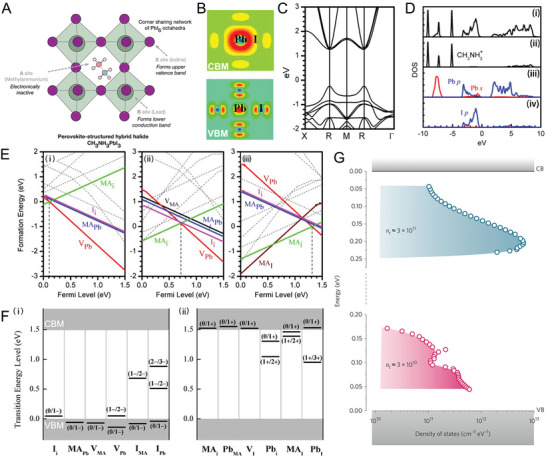
A) The crystal structure of MAPbI_3_. Reproduced with permission.^[^ ^40b^ ^]^ Copyright 2015, American Chemical Society. B) Partial charge density at CBM (upper) and VBM (lower), C) Band structure, and D) Density of states of MAPbI_3_, from (i) to (iv) are total DOS and MA^+^, Pb, I partial DOS respectively, E) The formation energies of intrinsic point defects in MAPbI_3_ as a function of Fermi level at three chemical potential points, from (i) to (iii) are I‐rich/Pb‐poor, moderate and I‐poor/Pb‐rich conditions, respectively. F) Calculated transition energy levels of intrinsic acceptors (i) and intrinsic donors (ii) of MAPbI_3_. Reproduced with permission.^[^ ^18^ ^]^ Copyright 2014, AIP Publishing. G) Density of the trap states of MAPbI_3_ single crystal extracted from temperature‐dependent SCLC technique. Reproduced with permission.^[^ ^43^ ^]^ Copyright 2016, Wiley‐VCH.

As the first breakthrough light‐absorbing MHP materials in the photovoltaic field,^[^ ^39^ ^]^ the crystalline and electronic structures of α‐phase MAPbI_3_ have been extensively studied with theoretical calculations as the prototypical model. In the crystalline structure of MAPbI_3_, the MA^+^ ions with larger sizes are weakly incorporated into the large voids of corner‐sharing PbI_6_ octahedra through van der Waals interactions (**Figure** **4**A).^[^ ^40^ ^]^ The electronic structure of MAPbI_3_ is mainly determined by the outer orbitals coupling of Pb and I ions. To be specific, the conduction band minimum (CBM) of MAPbI_3_ is mainly composed of the higher energy level Pb 6p orbital with modicum coupling with I 5p orbital, which results in the ionic character of the CB edge. Meanwhile, the electronic structure of valence band maximum (VBM) consisted of the strong interaction between Pb 6s orbital and I 5s orbital (Figure 4B–D).^[^ ^18^ ^]^ The original atomic orbitals are close to the band edges, hence the defect states (especially point defects) formed close to the original atomic orbitals mostly reside within the band rather than inside the bandgap, creating the shallow traps. It is worth noting that although the MA^+^ cation does not directly contribute to the electronic structure, the A‐site cations could tune the bandgap structure by distorting the Pb‐I framework through steric and Coulombic interactions, tilting the octahedral unit and modulating the overlapping of the coupling orbitals.^[^ ^41^ ^]^

As discussed above, 12 possible point defects of MAPbI_3_ have been screened.^[^ ^18^ ^]^ The defects with low formation energies such as MA_i_, V_Pb_, MA_Pb_, I_i_, V_I_, and V_MA_, have transition energies less than 0.05 eV above (below) VBM (CBM). The shallower characteristics of dominant acceptors such as V_Pb_ and MA_Pb_ originate from the antibonding state at the VBM, which lifts the VBM to a higher level so that these acceptors with sole I p orbital generally form shallower states. The primary donor states such as MA_i_ and V_i_ are due to the high ionicity of MA and Pb ions. Similar to MA cation, MA_i_ only provides one electron but does not form covalence bond within Pb‐I framework, thus generating no gap states. The CBM of MAPbI_3_ with the major contribution of Pb p orbital exhibits weak covalence, leading to shallow vacancy levels. The defects with deep transition energy levels (including I_Pb_, I_MA_, Pb_i,_ and Pb_I_) and responsible for non‐radiative recombination are thermodynamically unfavored, implying the nominal non‐radiative recombination rate of MAPbI_3_. Similar theoretical calculations have also been conducted on other common perovskite iodides FAPbI_3_^[^ ^36^ ^]^ and CsPbI_3_.^[^ ^42^ ^]^ Due to the same Pb‐I framework, the FAPbI_3_ and CsPbI_3_ exhibit similar band structures and point defect properties to MAPbI_3_ that deep level traps are less primary than the defects create shallow traps. Computational simulations have also been supported by experimental observations. The temperature‐dependent space charge‐limited current (SCLC) was conducted to extract the density of trap states (DOS_T_) of MAPbI_3_ single crystal and revealed that DOS_T_ localized only ≈0.2 eV from the conduction band and ≈0.1 eV from the valence band (Figure 4E–G), verifying the existence of shallow traps for both electrons and holes.^[^ ^43^ ^]^

**Figure 5:**
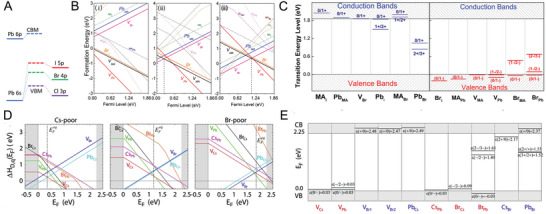
A) The construction of the CBM and VBM in MHP materials with different halogens. B) The formation energies of intrinsic point defects in MAPbBr_3_ as a function of Fermi level at three chemical potential points, from (i) to (iii) are Br‐rich/Pb‐poor, moderate, and Br‐poor/Pb‐rich conditions, respectively. C) The transition energy levels of intrinsic donors (left) and intrinsic acceptors (right) of MAPbBr_3_. Reproduced with permission.^[^ ^40a^ ^]^ Copyright 2022, IOP Publishing. D) Calculated defect formation energies in the CsPbBr_3_ as a function of Fermi level, from left to right are Cs‐poor, moderate, and Br‐poor conditions, respectively. E) Calculated transition energy levels for vacancies and anti‐site defects of CsPbBr_3_. Reproduced with permission.^[^ ^46^ ^]^ Copyright 2015. American Physical Society.

In addition to the triiodide compounds, bromide‐based MHPs have also been widely investigated, especially for photocatalytic applications. Analogues to the iodide perovskites, the band structures of APbX_3_ are mainly dependent on the Pb‐halogen framework. As mentioned above, the conduction bands are predominantly based on the fully empty Pb 6p orbital while the valence bands are constructed by the antibonding coupling between Pb 6s orbital and ns^2^ orbitals of halogens. The decrease in the energies of halogen ns^2^ orbitals and the shrinkage of the effective halogen atom radius in the order of I>Br>Cl widen the bandgap of perovskite halide (**Figure** **5**A) and diminish the antibonding coupling between Pb 6s orbital and halogen ns^2^ orbital in the same order, thus decreasing the bonding energy.^[^ ^44^ ^]^ The systematic computational studies on the point defect properties of MAPbBr_3_^[^ ^45^ ^]^ and CsPbBr_3_^[^ ^46^ ^]^ have also shown that the Br‐based perovskites possess intrinsic point defects distribution akin to the I‐based counterpart, as depicted in Figure 5B–E. Only the point defects involved with Pb‐Pb or Br‐Br bonding were expected to induce the deep level states and these defects are normally accompanied by great formation energies as well. Shi et al. found that Pb s and Br p antibonding coupling is stronger than that between Pb s and I p orbitals.^[^ ^45^ ^]^ The Pb‐Br antibonding coupling does not gain electronic energy, thus leading to the tendency of the Pb‐Br bond breaking and the lower formation energies of V_Pb_ and V_Br_ in MAPbBr_3_ than the corresponding defects in MAPbI_3_. Similarly, Liu et al. characterized the stable surface structure of MAPbBr_3_ with DFT calculations together with high‐resolution scanning tunneling microscopy (STM) simulation and revealed that the primary surface defects were V_Br_ and V_Br‐MA_,^[^ ^47^ ^]^ which have the lowest formation energies and introduce the electronic states close to the CBM. These surface vacant sites barely contribute to the non‐radiative recombination but increase the adsorption energies of H_2_O, O_2,_ and CH_3_CN molecules due to the hydrogen bonds between the adsorbates and defect sites, thus causing the possible structural decomposition of MAPbBr_3_.^[^ ^47^ ^]^

#### 2.2.2 Deep Level Defects

**Figure 6:**
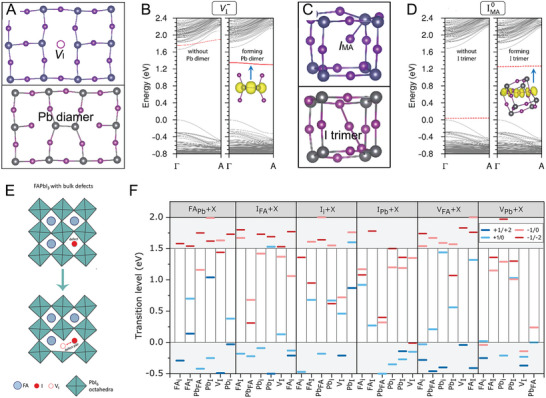
A) Atomic structure changes before (upper) and after (lower) the formation of a Pb dimer via V_I_. B) Band structure changes for V_I_ before (left) and after (right) the formation of Pb dimer. C) Atomic structure changes before (upper) and after (lower) the formation of an I trimer via I_MA_. D) Band structure changes for I_MA_ before (left) and after (right) the formation of I trimer. Reproduced with permission.^[^ ^50^ ^]^ Copyright 2014, American Chemical Society. E) Defect pair representation in FAPbI_3_. F) Calculated charge transition energy levels of defect pairs, the letter X in each box at the top represents one of the six donor defects to be paired with the corresponding acceptor defect in the same box. Reproduced with permission.^[^ ^53^ ^]^ Copyright 2022, American Chemical Society.

Although the MHPs are believed to be defect‐tolerant and more liable to accommodate the point defects that only introduce shallow traps, the defects with deep transition levels also inevitably exist and are regarded as the archcriminal for Shockley‐Read‐Hall (SRH) recombination centers. Despite the computational results suggested that the deep level traps normally have large formation energies, the oversimplified calculation conditions are far away from the practical growth environments of MHPs,^[^ ^48^ ^]^ let alone the substantial experimental evidence verifying the presence of the deep level traps in MHP‐based applications. Moreover, the formation energy of defects is also dependent on the MHP fabrication conditions and varied chemical compositions of MHPs have also given rise to the complicated defect generation processes.^[^ ^49^ ^]^ For instance, the I‐rich environment could endow a relatively low enough formation energy for Pb_I_ and I_MA_ anti‐site with the most stable charge state lying deep within the bandgap. In addition to the computational predicted intrinsic deep‐level point defects (such as Pb_I_, Pb_i_, I_MA_, I_Pb_), other species are also reported to generate the sub‐bandgap states in MHPs. With the first‐principal calculation, Agiorgousis and co‐workers revealed that the intrinsic defects (V_I_, Pb_i_, Pb_MA_, I_MA_) serve as the trigger of the formation of Pb dimers and I trimers due to the strong covalency of Pb cations and I anions (**Figure** **6**A–D).^[^ ^50^ ^]^ The stabilization of these intrinsic defects by the strong covalency at certain charge states begets the deep charge‐state transition levels within the bandgap, acting as the non‐radiative recombination centers. The formation of Pb dimers and I trimers associated with eight deep defect transition levels has also been obtained in α‐CsPbI_3_ by Wu et al. with molecular dynamic simulations.^[^ ^51^ ^]^ They found that two electrons in the conduction band and two holes in the valence band would induce the formation of a Pb dimer and an I trimer, respectively. Zhang et al. unveiled that the anti‐site defects Pb_I_ and I_Pb_ are energetically favorable and create states deep in the bandgap of CsPbI_3_.^[^ ^52^ ^]^ However, the CsPbI_3_ still has a low non‐radiative recombination rate ascribed to its strong anharmonicity, which can suppress the defect‐assisted non‐radiative recombination. In the case of FAPbI_3_, Liu et al. discovered that the FA‐related defects have low formation energies and the anti‐site defect of FA_I_ and I_FA_ stay deep within the bandgap of FAPbI_3_ and act as non‐radiative recombination centers with the reduced non‐equilibrium carrier lifetime,^[^ ^36^ ^]^ which is different from the MA_I_ anti‐site in MAPbI_3_. Sezen et al. studied the defect pair formation in FAPbI_3_ and uncovered that some donor and acceptor point defects individually are unstable and shallow (Figure 6E,F).^[^ ^53^ ^]^ However, they are possible to form stable and deep‐level defect pairs through mutual stabilization, such as I_i_ + Pb_I_, FA_Pb_ + FA_I_, I_i_ + FA_I_, V_Pb_ + FA_I_, V_Pb_ + Pb_i_, and FA_Pb_ + Pb_FA_.

Aside from the deep‐level point defects, the extended defects are more frequently investigated in experimental works considering they are more likely to emerge during fast crystal growth and post‐annealing processes and normally generate detrimental deep‐level states in the MHP thin films. For instance, the non‐stoichiometry of composition at the grain boundaries and interfaces brings about the undercoordinated ions.^[^ ^54^ ^]^ The evaporation of organic cations and halides can leave uncoordinated Pb^2+^ ions at the surface, which serve as the electron acceptor. Furthermore, the uncoordinated Pb^2+^ could be further reduced into the metallic Pb° clusters, which act as the donor states pinning the Fermi level at the surface of MHPs and causing the n‐type semiconducting character.^[^ ^55^ ^]^ Both uncoordinated Pb^2+^ and Pb^0^ were proved as deep defects for non‐radiative recombination and the presence of them was evidenced experimentally.^[^ ^56^ ^]^ The negatively‐charged uncoordinated halide anions and Pb‐I anti‐site (PbI_3_^−^) serve as the superficial donor at the grain boundaries or interface of MHPs, which could trap positive charges and holes in deep‐level states, thus delivering severe non‐radiative charge recombination and leading to the charge accumulation at the surface. The positively and negatively charged uncoordinated species could be regarded as Lewis acids and Lewis bases, respectively. Neutralization by constructing the corresponding Lewis adducts is the common approach for the passivation of charged defects. In particular, Lewis base can be used to coordinate with the uncoordinated Pb^2+^ through long pair electrons and therefore annihilate electronic trap states. Noel et al. initiated the application of Lewis base containing organic molecules (pyridine and thiophene) for the passivation of undercoordinated Pb^2+^ cation.^[^ ^57^ ^]^ The nitrogen on pyridine and sulfur on thiophene donate a pair of nonbonding electrons to coordinate with Pb^2+^. Due to the electron rich nature, the uncoordinated halide anions and PbI_3_^−^ are normally regarded as the Lewis bases, which could be passivated by introducing Lewis acid with the capability of accepting a pair of nonbonding electrons. Abate et al. first demonstrated the passivation of uncoordinated halide anions by a strong halogen bond donors iodopentafluorobenzene (IPFB).^[^ ^58^ ^]^ The strong electronegative fluorine atoms inductively withdraw electron density out of the aromatic ring of IPFB as well as the electron density from the iodine bonded to the aromatic ring, –C–I. The anisotropic distribution of positive electrostatic potential localized opposite the carbon on the extension of the −C–I bond direction. Therefore, the electron deficient iodine serves as the electron acceptor Lewis acid and interacts attractively with the electron‐rich uncoordinated halide anions or PbX_3_^−^ antisite defect, forming the strong –C–I···X bond for an enhanced charge transfer efficiency. Moreover, the uncoordinated I^−^ ions are susceptible to oxidization and followed by the generation of volatile I_2_. The release of I_2_ can cause the composition loss and thus irreversible decomposition of MHPs.^[^ ^59^ ^]^ Furthermore, the I_2_ vapor can provoke the degradation of MAPbI_3_ and other iodide‐based MHPs (such as FAPbI_3_ and FA_0.8_Cs_0.2_PbI_3_). In MAPbI_3_ thin film, the I_2_ could interact with mobile ions (I^−^ and MA^+^) and regenerate I_2_, followed by its participation in chemical reactions causing the further degradation of MAPbI_3_.^[^ ^60^ ^]^ Considering the accessible generation of I_2_ and the presence of mobile ions, the I_2_‐induced self‐degradation undoubtedly threatens the performance and long‐term stability of MHP‐based applications.

### 2.3 The Structures and Defect Properties of Pb‐Free MHP Derivates

**Figure 7:**
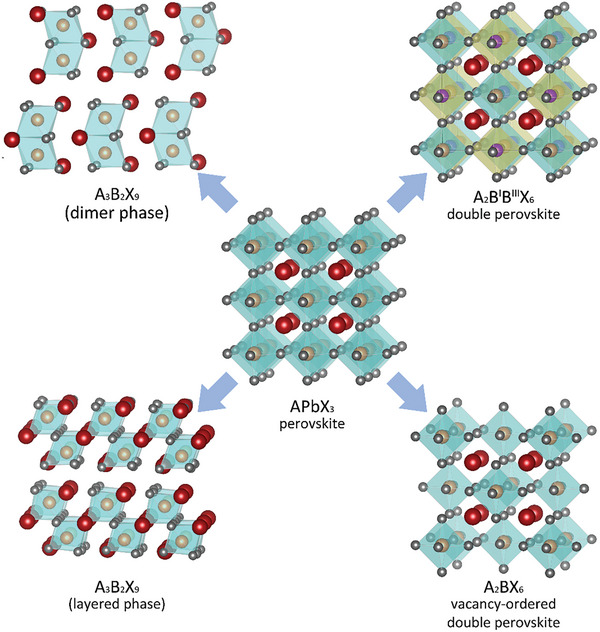
Structures of Pb‐based ABX_3_ perovskite and common lead‐free perovskite derivates.

Although Pb‐based MHPs exhibited superior advantages in different applications, the presence of the Pb element has raised widespread concern about its toxicity issues.^[^ ^61^ ^]^ Especially, in photocatalytic reactions where the unencapsulated MHP‐based catalysts are directly contacted with the liquid or gas medium, there is greater chance of the Pb leakage derived from the degradation of the Pb‐based MHP catalysts.^[^ ^62^ ^]^ Therefore, developing Pb‐free MHPs that possess similar outstanding attributes is of significant importance. Several alternatives have been screened to replace the Pb in APbX_3_ MHPs, including Sn, Ge, Bi, In, Sb, and Ag, to yield the Pb‐free MHPs.^[^ ^63^ ^]^ Substituting Pb^2+^ to its isovalent congeners from the same group (Sn and Ge) seems an ideal option because they have a similar outer‐shell electron configuration (ns^2^np^0^) with Pb and could meet the coordination and charge balance requirements, thus theoretically maintaining the traditional perovskite structure. Great endeavors have been made to explore the possibility of replacing Pb with Sn since the Sn‐based MHPs are expected with higher charge carrier mobility, greater theoretical solar energy conversion efficiency, and could circumvent the toxicity issue.^[^ ^64^ ^]^ However, the inherent instability coming from the readily oxidizable nature of Sn from divalent Sn^2+^ to tetravalent Sn^4+^ has caused severe p‐type self‐doping and thus badly restricted the performance of Sn‐based MHPs.^[^ ^65^ ^]^ The vulnerability to oxidation has also been reported on the Ge‐based MHPs.^[^ ^66^ ^]^ Therefore, attention has been directed to exploring the aliovalent substitutions of Pb^2+^. To keep the charge balance, the Pb‐free MHP derivates generally exhibit different substructures with varied stoichiometry compared to the traditional perovskite. In recent years, a series of Pb‐free MHP derivates have been developed (**Figure** **7**) and many of them have been demonstrated effective in photocatalytic applications. To deepen the understanding and guide the design of the Pb‐free MHP‐based photocatalysts, their crystalline and electronic structures and defect properties are reviewed as follows.

#### 2.3.1 A_3_B_2_X_9_ Type Derivates

**Figure 8:**
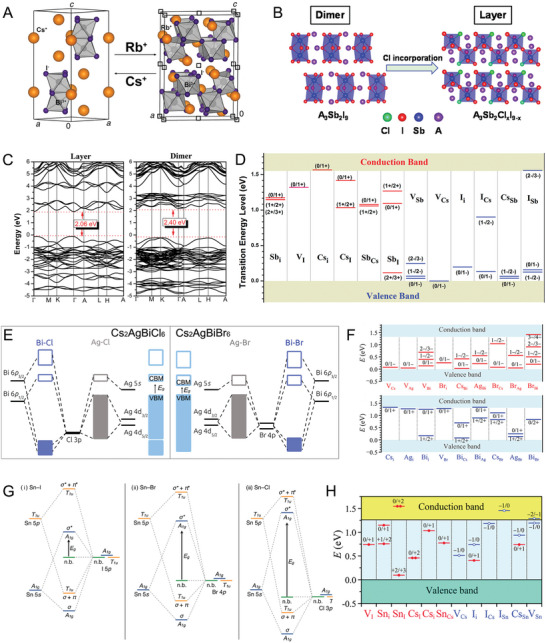
A) Crystalline phase variation of Cs_3_Bi_2_I_9_ (0D dimer phase) and Rb_3_Bi_2_I_9_ (2D layered phase). Reproduced with permission.^[^ ^69a^ ^]^ Copyright 2015, American Chemical Society. B) Cl‐induced phase transformation from the 0D dimer phase of A_3_Sb_2_I_9_ to the 2D layered phase of A_3_Sb_2_Cl*_x_* I_9–_ *_x_*. Reproduced with permission.^[^ ^70^ ^]^ Copyright 2018, American Chemical Society. C) Calculated band structures of Cs_3_Sb_2_I_9_ in layered and dimer modifications. D) Calculated transition energy levels of intrinsic donors (red lines) and acceptors (blue lines) in Cs_3_Sb_2_I_9_. Reproduced with permission.^[^ ^71^ ^]^ Copyright 2015, American Chemical Society. E) Schematic molecular orbital diagrams Cs_2_AgBiCl_6_ (left) and Cs_2_AgBiBr_6_ (right). The dark blue and the gray rectangles correspond to the Bi‐halide hybrid bands and the Ag‐halide hybrid bands, respectively. The light blue rectangles represent the bands formed in Cs_2_AgBiCl_6_ and Cs_2_AgBiBr_6_, respectively. Filled rectangles represent occupied (valence) bands and empty rectangles represent the unoccupied (conduction) bands. Reproduced with permission.^[^ ^80a^ ^]^ Copyright 2016, American Chemical Society. F) Calculated transition energy levels of intrinsic acceptors (upper) and donors (lower) in Cs_2_AgBiBr_6_. Reproduced with permission.^[^ ^81^ ^]^ Copyright 2016, Wiley‐VCH. G) The band structure of Cs_2_SnX_6_ predicted by the molecular orbital theory, (i) Cs_2_SnI_6_, (ii) Cs_2_SnBr_6_, and (iii) Cs_2_SnCl_6_. Reproduced with permission.^[^ ^82^ ^]^ Copyright 2019, American Chemical Society. H) Calculated transition energy levels for intrinsic defects in Cs_2_SnI_6_. Donor states are marked with red lines and acceptor states are denoted by blue lines. Reproduced with permission.^[^ ^88^ ^]^ Copyright 2015, Royal Society of Chemistry.

Replacing Pb^2+^ with the isoelectric ions such as Bi^3+^ and Sb^3+^ is a natural approach. For instance, Bi^3+^ has a similar 6s^2^6p^0^ outer‐shell electron distribution to Pb^2+^, which is crucial for the long carrier lifetime and sufficient light absorption.^[^ ^67^ ^]^ To meet the stable stoichiometry, a typical formula of A_3_B_2_X_9_ was obtained after changing the B‐site from divalent Pb^2+^ to trivalent Bi^3+^ or Sb^3+^. The A_3_B_2_X_9_ derivates commonly have two structural configurations: the 0D hexagonal phase featuring isolated [B_2_X_9_]^3–^ dimers with face‐sharing [BX_6_]^3–^ octahedra (Figure 7 upper left) and the 2D polymorph consisting corrugated layers of partially corner connected [BX_6_]^3–^ octahedra (Figure 7 bottom left). The latter one could be derived from the hypothetical cubic ABX_3_ crystalline structure by removing every third B‐layer along the ^[^ ^111^ ^]^ crystallographic direction.^[^ ^68^ ^]^ The dimensionality is related to the size of the ions. To be specific, for the case of A_3_Bi_2_I_9_, the Cs_3_Bi_2_I_9_ and MA_3_Bi_2_I_9_ show the 0D character but the ones with smaller A‐site cations (A = Rb^+^, NH^4+^, and K^+^) possess layered structures,^[^ ^69^ ^]^ as shown in **Figure** **8**A. Similarly, the A_3_Sb_2_I_9_ (A = Cs^+^ or MA^+^) present the 0D dimer phase but the incorporation of Cl anion could bring about the phase transformation to the 2D layered structures (Figure 8B).^[^ ^70^ ^]^

The different structural configurations have led to different electron structures. According to the DFT calculation results from Saparov et al.,^[^ ^71^ ^]^ the layered phase Cs_3_Sb_2_I_9_ has a nearly‐direct bandgap of 2.06 eV while a larger indirect bandgap of 2.40 eV was found for dimer phase Cs_3_Sb_2_I_9_ (Figure 8C), the difference in band gap has also been verified experimentally. In addition, the layered modification Cs_3_Sb_2_I_9_ has more dispersive CBM and VBM than the dimer phase Cs_3_Sb_2_I_9_, which implies better carrier transport properties. Both layered and dimer phases Cs_3_Sb_2_I_9_ have decent light absorption coefficients as high as that of MAPbI_3_, because of similar p‐p transitions between conduction and valence bands. In spite of the promising optical properties, the layered modification Cs_3_Sb_2_I_9_ exhibited a distinct defect property. The more localized p atomic orbital of Sb compared to that of Pb has led to the stronger Sb p‐I p antibonding, which accounts for the deeper donor defects levels in Cs_3_Sb_2_I_9_.^[^ ^71^ ^]^ Meanwhile, the slightly deeper acceptor states are partially caused by the weaker s‐p antibonding coupling in Cs_3_Sb_2_I_9_. As described in Figure 8D, most of the dominant point defects create deep level states within the bandgap except for V_Cs_ and Cs_i_. The presence of deep level states acts as the non‐radiative recombination center, confirmed by the greatly suppressed PL intensity with respect to that of MAPbI_3_. The 0D dimer Cs_3_B_2_X_9_ compounds show poor optoelectronic characteristics due to the lack of structural and electronic dimensionality. Ghosh et al. unveiled a similar intrinsic point defect distribution in the dimer phase Bi‐based ternary halide perovskite.^[^ ^72^ ^]^ In contrast to the MAPbI_3_, they found that point defects with low formation energies are also possible to create deep mid‐gap states in Cs_3_Bi_2_I_9_, which is regarded as the major issue for its poor solar energy conversion efficiency.

#### 2.3.2 Halide Double Perovskite Type Derivates (A_2_B^I^B^III^X_6_ and A_2_BX_6_)

Although replacing Pb^2+^ with trivalent Sb^3+^ and Bi^3+^ could reduce the environmental concern and achieve desirable stability, both 0D dimer and 2D layered phases have also reduced the dimensionality of 3D MHP structures, which gives rise to both the increase in the effective masses of charge carriers and the decrease in the lifetime of the charge carriers, as well as the reduction of the band dispersion.^[^ ^73^ ^]^ To fulfill the 3D structured MHP derivates, an emerging strategy with the B‐site replaced with a combination of one monovalent and one trivalent metal cations has been explored to form a cation‐ordered double perovskite structure with a typical formula of A_2_B^I^B^III^X_6_ (Figure 7 upper right).^[^ ^74^ ^]^ The structural configuration has offered numerous opportunities for A, B^I^, B^III^, and X sites.^[^ ^18^ ^]^ For the case of Cs‐based Cs_2_B^I^B^III^X, the monovalent B^I^ site is normally occupied by Ag^+^, Au^+^, Li^+^, Na^+^, and K^+^ while the trivalent cation such as Bi^3+^, Sb^3+^, In^3+^, and Tl^3+^ generally located at the B^III^ site.^[^ ^63^ ^]^

Several Cs_2_B^I^B^III^X_6_ candidates have been proven to possess the desired direct bandgap properties due to the unique outmost atomic orbital configurations, such as Cs_2_AgInCl_6_^[^ ^75^ ^]^ and Cs_2_InBiCl_6_,^[^ ^76^ ^]^ which is theoretically favorable for the light harvesting. However, these promising compounds suffer from their own dilemma such as the disallowed direct bandgap transition due to parity‐forbidden nature (Cs_2_AgInCl_6_)^[^ ^77^ ^]^ or the present unavailability for experimental preparation (Cs_2_InBiCl_6_). In addition to the above‐mentioned compounds with direct band gap but rather difficult to obtain, the Cs_2_AgBiX_6_ (X = Br and Cl) are extensively investigated among diverse halide double perovskites, due to the relatively small bandgap energy and decent stability.^[^ ^78^ ^]^ The Cs_2_AgBiX_6_ are indexed to the standard 3D cubic perovskite structure, which is constructed by the framework of orderly corner‐connected [AgX_6_]^5–^ and [BiX_6_]^3–^ octahedra and the Cs^+^ cations located in the voids of the framework.^[^ ^79^ ^]^ According to the calculation results, the band structures of Cs_2_AgBiX_6_ are determined by the [Ag‐X‐Bi] frameworks.^[^ ^80^ ^]^ As illustrated in Figure 8E, the VBM and CBM are mainly derived from the Ag 4d‐X np (n = 3 for Cl and n = 4 for Br) antibonding coupling and Bi 6p‐X np antibonding coupling states, respectively. The VBM is located at X point and the CBM is positioned at the L point of the Brillouin zone, respectively, causing the large indirect bandgap.^[^ ^80^ ^]^ The quaternary double perovskites are rational to have more types of intrinsic point defects compared to the APbX_3_ perovskite. 18 possible intrinsic point defects have been theoretically investigated and their transition energies are summarized in Figure 8F.^[^ ^81^ ^]^ Among the normal dominant defects with relatively lower formation energies, the V_Bi_ vacancy and Br_i_ interstitial act as the deep acceptor states within the bandgap. Moreover, out of the twelve anti‐sites, the B‐site cation‐on‐cation anti‐sites, especially the Ag_Bi_, are also proven to have relatively low formation energies as well as deep transition levels in the mid‐gap, taking the role of deep acceptor. Furthermore, because of mismatch between the Ag and Bi orbitals, the electronic dimensionality of Cs_2_AgBiBr_6_ is actually lower than 3D, which accounts for the calculated larger effective carrier masses of Cs_2_AgBiBr_6_.^[^ ^80^, ^81^ ^]^

Aside from the replacement of Pb^2+^ with trivalent cations or a couple of monovalent and trivalent cations, the B‐site substitution with tetravalent cations has also been demonstrated as an effective approach to achieving the stable and Pb‐free MHP derivatives, which generates the vacancy‐ordered double perovskite with a general formula of A_2_BX_6_ (Figure 7 bottom right). The structure of A_2_BX_6_ could be considered as a derivation from the ABX_3_ by doubling the parent unit cell along all three crystallographic axis and then removing the alternate cations at the B‐site (ABX_3_→A_2_B_2_X_6_→A_2_BX_6_).^[^ ^82^ ^]^ The removed B‐site vacancies are also marked as □ so the A_2_BX_6_ variants are sometimes denoted as A_2_B□X_6_. Considering the absence of [BX_6_]^2–^ octahedra connectivity, the A_2_B□X_6_ is viewed as quasi‐0D perovskite transmutation,^[^ ^41a^ ^]^ which is similar to the dimer phase Cs_2_Bi_3_X_9_.

Several tetravalent cations have been screened to accommodate the B‐site, including Sn, Te, Ti, Zr, Pt, and Pd.^[^ ^83^ ^]^ A recent work by Folgueras et al. even integrated six different tetravalent cations into the B‐site of Cs_2_BCl_6_ crystalline lattice,^[^ ^84^ ^]^ indicating the excellent formability and compatibility of such structure. Among dozens of Cs_2_BX_6_ compounds, the Cs_2_SnX_6_ has stood out and been spotlighted in the photovoltaic and photocatalytic fields due to the suitable bandgap of 1.3 eV (for Cs_2_SnI_6_) and good air‐stability.^[^ ^85^ ^]^ As opposed to the ASnX_3_ MHPs, the higher oxidation state of tin in Cs_2_SnX_6_ compounds makes them more robust against the oxidative degradation. Due to the isolation of [SnX_6_]^2–^ octahedra in Cs_2_SnX_6_, these double perovskites exhibit quite different electronic properties from their 3D CsSnX_3_ counterparts. In contrast to the antibonding nature of the VBM of CsSnX_3_, the VBMs of Cs_2_SnX_6_ consist of the nonbonding states of the halogen p orbital (Figure 8G).^[^ ^82^ ^]^ The VBM is localized and quite flat along the Γ‐X direction, leading to a large effective hole mass (m^*^_h_) and therefore poor hole mobility along this direction. The CBMs of Cs_2_SnX_6_ are derived from the antibonding coupling of Sn 5s and halogen orbitals. The large conduction band dispersion due to the dispersed nature of Sn 5s states contributes to the smaller effective electron mass (m^*^_e_) than its m^*^_h_ and thus the moderate electron mobility. It is also important to note that although some Cs‐based A_2_BX_6_ perovskite derivates theoretically have the fundamental direct bandgap, defined as the energy difference between CBM and VBM, may not reflect the actual optical absorption onset obtained from the ultraviolet‐visible spectroscopy. For example, the Cs_2_SnI_6_ is characterized by a direct band gap semiconductor with its VBM and CBM at the Γ point.^[^ ^86^ ^]^ The transition from its VBM to CBM is dipole‐forbidden because of the inversion symmetry.^[^ ^75b^ ^]^ Therefore, the dominant optical bandgap arises from the states lower than the valence band edge rather than the VBM, causing a wider optical band gap than its fundamental band gap. Similar cases have also been demonstrated on Cs_2_TiBr_6_^[^ ^84^ ^]^ and Cs_2_PdBr_6_,^[^ ^87^ ^]^ whose direct bandgap transitions are dipole‐disallowed as well. In addition to the optical properties, the systematic theoretical calculation work has revealed that the Cs_2_SnX_6_ is less defect tolerant than the 3D CsSnX_3_. Xiao et al. evaluated the electronic structures of Cs_2_SnI_6_^[^ ^88^ ^]^ and found that the removal of half Sn and the subsequent isolation of [SnI_6_]^2–^ octahedra change the energies of Sn 5s‐I 5p bonding and antibonding coupling states. Therefore, some interesting point defects that are reported shallow in CsSnI_3_ become deep level defects in Cs_2_SnI_6_, such as V_Sn_ and V_I_. Besides, because of the strong Sn‐I covalent bonds in the [SnI_6_]^2–^ clusters, the dominant intrinsic point defects (Cs_i_, Sn_i_, V_I_, V_Cs_) lie deeply in the band gap as shown in Figure 8H, working as the electron and hole trap centers. Likewise, other vacancy‐ordered double perovskites such as Cs_2_TeI_6_,^[^ ^89^ ^]^ Cs_2_TiI*_x_* Br_6–_ *_x_* ^[^ ^90^ ^]^ and Cs_2_PdBr_6_^[^ ^91^ ^]^ have also been demonstrated to present dominant intrinsic point defects with deep level states, causing the decrease in their charge carrier concentration and mobility as well as the non‐radiative recombination. The favorable stability and environmental benignity of the Pb‐free Cs‐based vacancy‐ordered double perovskites have captured great attention for exploring their possibility in photocatalytic applications. Recently, Ye et al. computationally screened dozens of Cs_2_BX_6_ compounds and pointed out some candidates (Cs_2_TeI_6_, Cs_2_TiBr_6_, Cs_2_SnBr_6_, and Cs_2_PtBr_6_) with the bandgap greater than 1.6 eV are promising in photocatalytic water splitting since their band edge alignments meet the water redox potential.^[^ ^92^ ^]^ These findings highlight the rational design of Pb‐free MHP derivates for the high‐performance photocatalysts.

The influence of defects of MHPs has been intensively explored on their application focusing on solar cells and LEDs. Most of the defects are recognized as detrimental or harmless at best. It is worth noting that the photocatalytic process is born with distinct working principles and mechanisms with these photovoltaic or optoelectronic devices. Therefore, it is rational to speculate that the roles that defects play in photocatalysis might be divergent and peculiar. For instance, some shallow defects in MHP photocatalysts might expand the light absorption range of photocatalysts or facilitate the absorption of the reactants without compromising the overall photocatalytic performance, just like the defects that have been performed in the traditional photocatalysts.^[^ ^38^ ^]^ Regarding A_3_B_2_X_9_ derivatives, A_2_B^I^B^III^X_6,_ and A_2_BX_6_ MHP materials, point defects are still the primary defects due to the low formation energy. Taking their typical compound Cs_3_Bi_2_Br_9_, Cs_2_AgBiBr_6_, and Cs_2_SnBr_6_ as examples, Br vacancies are prone to form during the solution processing. However, the point defects in A_3_B_2_X_9_ make it possible to create deep mid‐gap states within the bandgap except for V_A_ vacancy and A_i_ interstitial, which could act as the non‐radiative recombination center. By contrast, V_X_ vacancy, X_i_ interstitial, and B^I^_B_^III^ anti‐site in A_2_B^I^B^III^X_6_ usually act as the deep acceptor states within the bandgap, taking the role of the deep acceptor, while dominant point defects like A_i_, B_i_, V_X_, and V_A_ generally lie deeply in the band gap due to the strong B‐X covalent bonds in the [BX_6_]^2–^. In the following part, the effects of defects in different steps of photocatalysis will be discussed.

## 3 Beneficial Effects of Defects in Photocatalysis

Defects exist in almost all semiconductor materials. Although defects in semiconductors are widely recognized as charge recombination centers being detrimental to photocatalytic performance, more and more studies have shown that precisely engineered certain types of defects with suitable amounts can improve the photocatalytic performance of photocatalysts (TiO_2_, g‐C_3_N_4_, In_2_O_3_, etc.).^[^ ^38^ ^]^ These defects optimize the electronic structure, thereby tuning the light absorption, influencing the charge separation, and offering more reactive sites. For MHP materials, so far, most studies still consider the defects harmful for photocatalytic applications, while some recent research has demonstrated the positive effects of defects that contribute to photoactivity enhancement. Thus, in this section, we will focus on highlighting the positive roles of defects in MHP materials for photocatalytic solar‐to‐chemical conversion, analogous to some typical traditional photocatalysts.

### 3.1 Light Absorption

As is known, the light absorption of semiconductors depends on the bandgap which is associated with the electronic structure of the materials, where the electronic structure can be modulated by the introduction of defects (vacancies, dopants, lattice disorders, etc.). In general, the defect sites in semiconductor materials possess weaker bonding.^[^ ^38^ ^]^ Compared to the valence and conduction band states, the weaker bonding can reduce the splitting between bonding and antibonding orbitals, yielding the electronic states within the bandgap. As a result, the generated electronic states can be favorable for narrowing the bandgap of the materials or serving as midgap states for the photogenerated charge carriers, thereby extending the light absorption range of the photocatalyst. Thus, defect engineering has been adopted to improve the visible light absorption of the photocatalyst.

**Figure 9:**
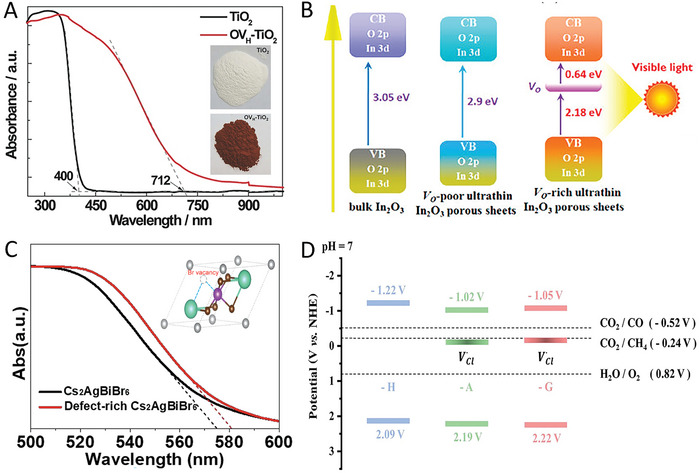
A) UV‐vis absorption spectra of TiO_2_ with and without hydrogen‐mediated oxygen vacancies (O_VH_). Inset shows the digital photos of the materials. Reproduced with permission.^[^ ^93^ ^]^ Copyright 2018, Wiley‐VCH. B) Schematic illustration of the band structure of In_2_O_3_ and V_O_‐rich In_2_O_3_. Reproduced with permission.^[^ ^94^ ^]^ Copyright 2014, American Chemical Society. C) UV‐vis absorption spectra of Cs_2_AgBiBr_6_ and Br defect‐rich Cs_2_AgBiBr_6_. Reproduced with permission.^[^ ^11e^ ^]^ Copyright 2021, American Chemical Society. D) Band edge positions of Cs_2_NaBiCl_6_ with and without Cl vacancies. Reproduced with permission.^[^ ^11a^ ^]^ Copyright 2022, Wiley‐VCH.

For instance, Yang et al. reported that by introducing atomic hydrogen‐mediated oxygen vacancies (O_VH_),^[^ ^93^ ^]^ a red TiO_2_ (O_VH_‐TiO_2_) with strong visible light absorption was achieved, where the absorption edge is beyond 700 nm compared with that of 400 nm in traditional anatase TiO_2_ (**Figure** **9**A). Xie and co‐workers developed an oxygen‐vacancy‐rich ultrathin porous In_2_O_3_ photocatalyst.^[^ ^94^ ^]^ The resultant V_O_‐rich In_2_O_3_ exhibited a narrowed bandgap from 3.05 to 2.82 eV compared with the bulk In_2_O_3_ (Figure 9B). Yu et al. reported that in‐situ introduced nitrogen vacancies,^[^ ^95^ ^]^ significantly redshift the absorption edge of g‐C_3_N_4_, resulting in the bandgap change from 2.68 to 2.36 eV. Beyond these materials, adopting defect engineering to improve the light absorption of photocatalytic materials was also reported in WO_3_,^[^ ^96^ ^]^ Bi_2_WO_6_,^[^ ^97^ ^]^ SrTiO_3_,^[^ ^98^ ^]^ and BiOCl^[^ ^99^ ^]^ systems.

Moving to MHP materials, similar phenomena were also observed in MHPs. For instance, He et al. reported a light irradiation‐induced defect‐rich Cs_2_AgBiBr_6_ material via inducing the surface Br defects.^[^ ^11e^ ^]^ Since the change of local atomic arrangement and electronic structure in Cs_2_AgBiBr_6_, the absorption edge of the Cs_2_AgBiBr_6_ with Br defects exhibited a slight red shift, with the narrowed bandgap from 2.02 to 1.98 eV (Figure 9C). Pi et al. synthesized a 3D hierarchical chlorine‐vacancy rich Cs_2_NaBiCl_6_,^[^ ^11a^ ^]^ declining the bandgap from 3.31 to 3.21 eV (Figure 9D). Chen et al. showed that the Br vacancy engineering in Cs_3_Bi_2_Br_9_ resulted in the bandgap change from 2.54 to 2.27 eV.^[^ ^100^ ^]^ Recently, the defect engineering for tunning the optical properties of MHPs has also been demonstrated in MAPbI_3_,^[^ ^11b^ ^]^ CsPbBr_3_,^[^ ^101^ ^]^ and Cs_3_Sb_2_I_9_.^[^ ^11c^ ^]^ Note that accurately modulating the defect concentration and location in MHPs during the synthesis process is still difficult, and also the defect engineering cannot guarantee a narrowed bandgap or extended absorption range. On the other hand, considering that the photocatalytic performance of a photocatalyst is determined by multiple factors, we should clearly know that enhanced light absorption doesn't ensure improved photoactivity. Some other import factors, i.e., charge dynamics and surface reactive sites, should also be taken into account together.

### 3.2 Charge Dynamics

It is well‐documented that the acceleration of a photoredox reaction is driven by the photogenerated electrons and holes of the photocatalyst. Under light irradiation, the photocatalyst absorbs incident photons with enough energy to generate electron‐hole pairs. To achieve high photocatalytic performance, the photogenerated charge carriers need to be separated and migrated efficiently from bulk to surface to participate in the chemical reaction. Although some types of defects or excessive defects may act as charge recombination centers, it has been shown that the charge transfer and separation in photocatalysts can be promoted in the defective photocatalysts.^[^ ^10c^ ^]^ This is because that the defect‐induced electronic states can influence charge carrier dynamics, which offers a route for the energetic relaxation of charge carriers within the valence or conduction band, and this has been widely reported in traditional semiconductor materials.

**Figure 10:**
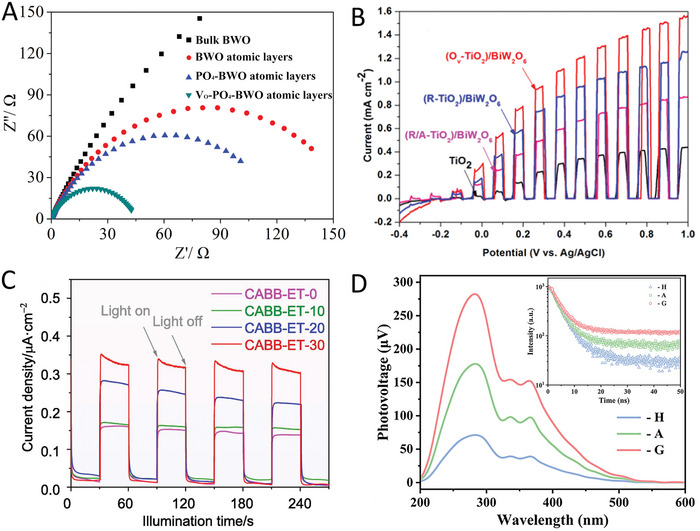
A) Electrochemical impedance spectra of Bi_2_WO_6_ (BWO) and V_O_‐PO_4_‐BWO. Reproduced with permission.^[^ ^102^ ^]^ Copyright 2016, Elsevier. B) Linear sweep voltammetry curves of TiO_2_ and defect‐engineered TiO_2_ in the TiO_2_/Bi_2_WO_6_ systems. Reproduced with permission.^[^ ^103^ ^]^ Copyright 2017, Elsevier. C) Transient photocurrent response of Cs_2_AgBiBr_6_ and the samples with Cs vacancies. Reproduced with permission.^[^ ^11f^ ^]^ Copyright 2022, Elsevier. D) Surface photovoltage of Cs_2_NaBiCl_6_ and Cl‐vacancy rich Cs_2_NaBiCl_6_ samples. Reproduced with permission.^[^ ^11a^ ^]^ Copyright 2022, Wiley‐VCH.

For instance, Hou and co‐workers synthesized a phosphate (PO_4_) and oxygen‐vacancy (V_O_) confined in Bi_2_WO_6_ (BWO).^[^ ^102^ ^]^ Electrochemical impedance spectroscopy (EIS) measurements showed a reduced resistance in V_O_‐PO_4_‐BWO (**Figure** **10**A), suggesting the efficient change transfer and separation in the sample. Bai et al. showed that oxygen vacancies in TiO_2_ are beneficial for charge separation,^[^ ^103^ ^]^ where the defect‐engineered TiO_2_ in the TiO_2_/Bi_2_WO_6_ hybrid exhibited a higher photocurrent density compared to the pristine one (Figure 10B). These studies showed that the precisely controlled defects are beneficial for charge separation, which is also confirmed by some other materials, such as BiOCl,^[^ ^99^ ^]^ g‐C_3_N_4_,^[^ ^104^ ^]^ and ZrO_2_.^[^ ^105^ ^]^

Inspired by these encouraging studies, more and more researchers have also started to reevaluate the effect of defects on the charge dynamics of MHPs and ultimately photocatalytic performance. Impressively, some recent studies have shown that small amounts of defects are beneficial for boosting the photocatalytic reaction. For example, Zhang et al. synthesized a Cs_2_AgBiBr_6_ sample with surface Cs vacancies through a supersaturated crystallization strategy.^[^ ^11f^ ^]^ During the synthesis process, the strong interaction between the chelating agent EDTA and Cs^+^ ion leads to the generation of Cs vacancies of Cs_2_AgBiBr_6_. Under light irradiation, the vacancy‐rich sample exhibited a higher photocurrent compared to the original Cs_2_AgBiBr_6_ (Figure 10C), suggesting the suppressed charge recombination and fast electron transfer efficiency. This is collaborated by other characterizations such as PL and EIS. A similar result was also reported by Pi et al.^[^ ^11a^ ^]^ They synthesized a Cs_2_NaBiCl_6_ material with Cl vacancies and studied the charge separation and transfer behavior of the sample. The surface photovoltage test results showed a larger photovoltage response for Cl‐vacancy Cs_2_NaBiCl_6_ (Figure 10D), along with an increased carrier lifetime (inset in Figure 10D), indicating the formed defects are favorable for the separation of photogenerated electron‐hole pairs. Together with PL, EIS, and Tafel plots measurements, they confirmed the superiority of Cl vacancy rich Cs_2_NaBiCl_6_ in charge transfer. Also, CsPbBr_3−_ *_x_* I*_x_* with I vacancies,^[^ ^106^ ^]^ Cs_3_Sb_2_I_9_ with I vacancies,^[^ ^11c^ ^]^ Cs_2_AgBiBr_6_ with Br vacancies,^[^ ^11d,e,g^ ^]^ Cs_3_Bi_2_Br_9_ with Br vacancies,^[^ ^100^ ^]^ have been discovered to facilitate the charge separation and transfer. Therefore, deliberately designing and synthesizing defects in MHP materials is promising for booting charge dynamics.

### 3.3 Surface Reaction

Apart from light absorption and charge dynamics, faster surface reaction kinetics are also important for achieving high photocatalytic performance. Since the semiconductor‐based photocatalytic reactions take place on the surface of the photocatalysts, defect engineering could be a useful strategy to promote the surface reaction kinetics of the photocatalysts from the below aspects:^[^ ^38^, ^107^ ^]^

i) Surface defects are associated with the dangling bonds or unsaturated atoms which are thermodynamically unstable and may be beneficial for the adsorption and activation of reactant molecules.^[^ ^38^ ^]^
ii) Some point defects like surface vacancies can act as the active site to directly involved in surface reactions;^[^ ^10^, ^38^ ^]^ meanwhile, the charge carriers localized at the surface defects can also promote the activation of the adsorbed reactant molecules.^[^ ^10b^ ^]^
iii) The positively or negatively charged surfaces can facilitate the adsorption of reactants with opposite charges via the electrostatic interaction.^[^ ^107b^ ^]^
iv) Surface defects may change the reaction pathways of the reactants, which improves the selectivity of the photoredox reactions.^[^ ^10^, ^38^ ^]^

To sum up, defect engineering is a feasible strategy to modify the surface chemistry and thus improve the photocatalytic performance of the catalysts. For MHP‐based photocatalysis, the effect of defects on the surface reaction, for example the adsorption energy of reactant and Gibbs free energy, has been investigated in some recent studies, which will be discussed in the next section.

## 4 Recent Progress in Defect Engineering

**Figure 11:**
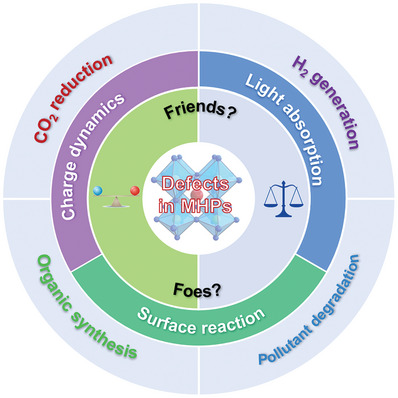
The effect of defects on MHP materials and the applications of defect‐contained MHPs on various photoredox systems.

Recently, MHPs have been shown to be promising photocatalysts for various photoredox applications. However, the role of the defects in MHP‐based photocatalysis is controversial. More specifically, some researchers showed that the defects compromise the photoactivity,^[^ ^8^, ^108^ ^]^ and they proposed that defect passivation can improve the photocatalytic performance of MHP materials;^[^ ^8a^ ^]^ while some others claimed that the defects are beneficial for the photoredox reactions, and the rational design of defects has been demonstrated to improve the photocatalytic activity of MHPs.^[^ ^11^ ^]^ Herein, we summarize the recent progress of two reverse defect engineering strategies, i.e., amending the surface defects or deliberately creating defects, for boosting MHP‐based photocatalysis (**Figure** **11**), mainly focusing on discussing and appraising the utilization of defects for photocatalytic applications.

### 4.1 Negative Effect of Defects

One of the main advantages of MHP materials is that they can be synthesized easily through solution‐processing, while this process can generally cause defects formation. Specifically, the defects are generally divided into two categories: intrinsic and extended defects.^[^ ^4b^ ^]^ Intrinsic point defects include vacancies, interstitials, and anti‐sites (Figure 2), with an increasing formation energy. Note that the defects in MHPs generally lie near the band edges or within the bands, making MHPs generally defect‐tolerant (Figure 2) compared to traditional defect‐intolerant semiconductors (e.g., CdSe, GaAs) (Figure 1A). Extended surface defects, originating from unsaturated surface bonds or the surrounding environment, are a major concern in solution‐processed MHPs.^[^ ^4b^ ^]^ Among these, halogen vacancies (V_X_) are the dominant defects on most MHP nanocrystals.^[^ ^8a^ ^]^ The existence of V_X_ would act as the charge recombination centers and slow down the charge transfer efficiency during the photocatalysis. To address this issue, passivation strategy for MHP has been widely adopted. Here, some typical examples are shown and discussed below.

**Figure 12:**
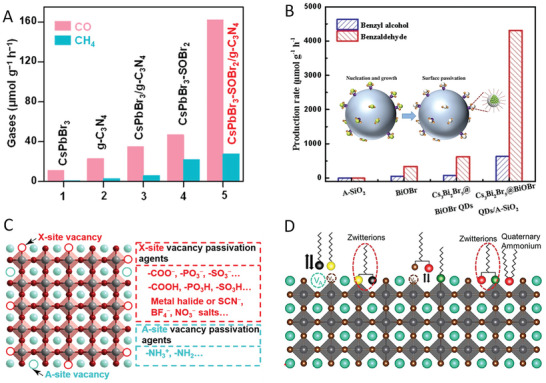
A) Photocatalytic performance of CO_2_ reduction over CsPbBr_3_, Br‐rich CsPbBr_3_, and their composites with g‐C_3_N_4_. Reproduced with permission.^[^ ^8c^ ^]^ Copyright 2022, American Chemical Society. B) Photocatalytic performance of toluene oxidation over Cs_3_Bi_2_Br_9_, Cs_3_Bi_2_Br_9_@BiOBr, and Cs_3_Bi_2_Br_9_@BiOBr/A‐SiO_2_. Reproduced with permission.^[^ ^8d^ ^]^ Copyright 2022 Wiley‐VCH. C) Summary of ligand modification of MHPs with different types of passivation ligands. Reproduced with permission.^[^ ^8a^ ^]^ Copyright 2022, American Chemical Society. D) Schematic illustration of defects passivation with various surface ligands. Reproduced with permission.^[^ ^112^ ^]^ Copyright 2021, Wiley‐VCH.

Zhang et al. reported that the surface halogen compensation on CsPbBr_3_ with SOBr_2_ was effective for the Br vacancies (V_Br_) preparation.^[^ ^8c^ ^]^ When applied to photocatalytic CO_2_ reduction, the Br‐filled surface of CsPbBr_3_ nanocrystals prohibited the undesired charge recombination, CsPbBr_3_‐SOBr_2_ shows an enhanced photocatalytic activity toward CO_2_ reduction (69 µmol g^−1^ h^−1^ for CO and CH_4_), which is about 6 times higher than that of the pristine CsPbBr_3_ (**Figure** **12**A). Moreover, when coupling CsPbBr_3_‐SOBr_2_ sample with g‐C_3_N_4_, such a halogen compensation method facilitates the charge transfer from CsPbBr_3_ NCs to nearby g‐C_3_N_4_, resulting in a high CO production rate of 190 µmol g^−1^ h^−1^ in photocatalytic CO_2_ reduction. A similar approach was also adopted by Lou and coworkers.^[^ ^8d^ ^]^ They coated the BiOBr on the surface of Cs_3_Bi_2_Br_9_ to achieve the passivation of surface defects in Cs_3_Bi_2_Br_9_ within the A‐SiO_2_. The experimental characterizations showed that the effective passivation of surface defects suppresses the non‐radiative recombination and thus facilitates the utilization of photogenerated charge carriers in the catalytic reaction. As a result, the Cs_3_Bi_2_Br_9_@BiOBr/A‐SiO_2_ showed a conversion rate of 4317 µmol g^−1^ h^−1^) for the selective oxidation of toluene to benzaldehyde (Figure 12B). Following the above reports, passivating the MHP defects to enhance the photocatalytic performance has been widely reported in various MHP systems, such as CsPbX_3_ (X = Cl, Br, I),^[^ ^8^, ^109^ ^]^ MAPbBr_3_,^[^ ^110^ ^]^ Cs_2_AgSbCl_6_,^[^ ^108^ ^]^ and Cs_3_Sb_2_(Br*_x_* I_1−_ *_x_*)_9_.^[^ ^111^ ^]^

Overall, at this stage, defect passivation is still the mainstream approach being widely used for MHP‐based photocatalytic systems (Figure 12C). Especially, post‐treatment with proper ligand molecules or metal salts has been employed to passivate the surface defects (Figure 12D). While this is not the focus of this review, interested readers are encouraged to explore the mentioned literature.^[^ ^8^, ^113^ ^]^ On the contrary, it is worth mentioning that the defects may serve as catalytic sites for adsorbing target reactants or even directly involved in surface reactions toward photocatalysis. Therefore, better understanding the relationship between the defects (especially surface defects) and catalytic activity and balance the pros and cons of defects is another interesting topic in photocatalysis, which is largely unexplored for MHP photocatalysts.

### 4.2 Positive Effect of Defects

So far, MHPs have been successfully used for various photocatalytic reactions, such as CO_2_ reduction, H_2_ evolution, organic synthesis, and pollutant degradation. In this section, recent progress in the development of defective MHP‐based composites on these reaction systems will be summarized and discussed, with some typical examples.

#### 4.2.1 CO_2_ Reduction

Inspired by the photosynthesis of plants, converting CO_2_ into value‐added chemicals (CO, CH_4_, CH_3_OH, etc.) with solar light has attracted much attention.^[^ ^8a^ ^]^ As a promising approach, photocatalytic CO_2_ reduction not only reduces CO_2_ emissions but also converts them into fuels that fulfill the criteria for a carbon‐neutral recycling process. Generally, the photoreduction of CO_2_ involves three key steps:^[^ ^1^, ^114^ ^]^ (1) light‐harvesting and charge carrier generation in semiconductor photocatalysts, (2) charge separation and migration from bulk to the surface of the photocatalysts, and (3) CO_2_ adsorption and product desorption on semiconductors surface. Since the pioneering work performed by Xu et al. using CsPbBr_3_ as the photocatalyst,^[^ ^115^ ^]^ MHPs have been demonstrated to be capable of driving photocatalytic CO_2_ reduction. However, the photocatalytic activity is still unsatisfactory mainly due to inefficient charge separation and transfer, sluggish surface reaction, and undefined reactive sites,^[^ ^7^, ^9^ ^]^ which limits their further applications. To boost photoactivity, enormous efforts have been made on cocatalyst loading and heterojunction construction.

Recently, defect engineering on MHP photocatalysts has been reported experimentally and/or theoretically beneficial for boosting the photocatalytic CO_2_ reduction process.^[^ ^11^, ^116^ ^]^ Note that defects commonly exist in materials. It's true that excess defects in semiconductors can act as charge recombination centers, while some studies have shown that when precisely engineered certain types of defects can improve the performance of the photocatalysts, where the defects optimize the electronic structure, tune the light absorption, influence the charge separation, and offer more reactive sites.^[^ ^38^ ^]^ Following this concept, defect engineering of MHPs, especially the halogen‐associated surface regulation, can be promising for the photocatalytic CO_2_ reduction since it offers effective regulation on states of surface atoms and active site configuration to improve the photophysical properties and reaction activity.

**Figure 13:**
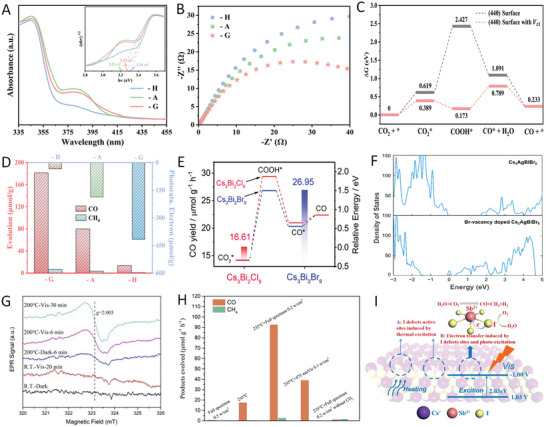
A) Absorption spectra, B) Electrochemical impedance plots, C) Gibbs free energy profiles, and D) Photocatalytic performance of CO_2_ reduction over Cs_2_NaBiCl_6_ (‐H), chlorine vacancy Cs_2_NaBiCl_6_ (‐A) and Cs_2_NaBiCl_6_ (‐G), where H, A, and G represent the Cs_2_NaBiCl_6_ prepared using hydrothermal, annealing and grinding method. Reproduced with permission.^[^ ^11a^ ^]^ Copyright 2022, Wiley‐VCH. E) The relationship between photoreduction CO_2_ to CO and reduction free energy of the main reactions in the photocatalytic CO_2_ reduction processes over Cs_3_Bi_2_Br_9_ and Cs_3_Bi_2_Cl_9_ photocatalysts. Reproduced with permission.^[^ ^118^ ^]^ Copyright 2020, American Chemical Society. F) Density of states plots of Cs_2_AgBiBr_6_ with and without Br‐vacancy. Reproduced with permission.^[^ ^116^ ^]^ Copyright 2021, The authors, American Chemical Society. Published by MDPI. G) In‐situ EPR data of Cs_3_Sb_2_I_9_ in toluene under light irradiation. H) The photocatalytic performance of CO_2_ reduction over Cs_3_Sb_2_I_9_ under photo‐, thermo‐, and photothermal synergistic catalysis. I) Schematic illustration of CO_2_ reduction over Cs_3_Sb_2_I_9_ catalyst with the photothermal synergistic effect. Reproduced with permission.^[^ ^11c^ ^]^ Copyright 2021, Elsevier.

For instance, Pi et al. synthesized a Cl‐vacancy Cs_2_NaBiCl_6_ sample for photocatalytic CO_2_ reduction.^[^ ^11a^ ^]^ As shown in **Figure** **13**A, the Cs_2_NaBiCl_6_ with Cl vacancy (Cs_2_NaBiCl_6_‐G) shows a stronger light absorption with a decreased bandgap. EIS plots showed the radius of Cs_2_NaBiCl_6_‐G much smaller than that of Cs_2_NaBiCl_6_ (Figure 13B), suggesting the effective charge transfer. Besides, Gibbs free energy profiles revealed that CO_2_ reduction on Cs_2_NaBiCl_6_‐G is more favorable in thermodynamics (Figure 13C), where the decreased free energy reduces the activation energy barrier and accelerates the formation of intermediate species. As a result, the photocatalytic CO_2_ reduction activity over Cs_2_NaBiCl_6_‐G enhanced 12.3 times compared to that of pristine Cs_2_NaBiCl_6_ (Figure 13D). In a similar fashion, Geyer et al. reported that Cs_3_Sb_2_Br_9_ with predominate Br defects significantly improved photocatalytic CO yield.^[^ ^117^ ^]^ They calculated the surface energies and adsorption free energies of the Cs_3_Sb_2_Br_9_ surface and found that highly exposed Sb sites resulted in lower free energy for the adsorption of COOH^*^ and CO^*^ intermediates, contributing to the enhanced catalysis activity. The beneficial effect of Br vacancies in MHPs for photoreduction of CO_2_ was further confirmed by Yan and coworkers.^[^ ^11g^ ^]^ Combined DFT calculations and experimental characterizations, they showed that surface Br vacancies in Cs_2_AgBiBr_6_ promoted the adsorption/activation of CO_2_ molecules and reduced the formation energy barrier of intermediates, resulting in a 30 times higher CH_4_ production rate (22.6 µmol g^−1^ h^−1^) than that of pristine Cs_2_AgBiBr_6_ in H_2_WO_4_/Cs_2_AgBiBr_6_ system. In addition, Shyamal et al. also showed that Br‐deficient dim multifaceted CsPbBr_3_ can enhance the photoactivity of CO_2_ reduction.^[^ ^101^ ^]^ While the Br vacancies in this work were deduced from the photoluminescence quantum yield measurements, more evidence for the existing vacancy should be further explored.

Although these studies showed the beneficial effect of defects, the role of the surface halogen‐associated active sites and reaction intermediates mediated by surface defects was not studied thoroughly. Given this, Sheng et al. investigated the impact of surface defect sites of Cs_3_Bi_2_X_9_ (X = Cl and Br) on photocatalytic CO_2_ reduction (Figure 13E),^[^ ^118^ ^]^ in which the surface halogen regulation effects were dynamically monitored and precisely unraveled combined in situ DRIFTS and DFT calculations. It was found that the surface Br sites can effectively narrow the bandgap, suppress the charge carrier recombination, and promote directional electron delivery. More importantly, it can alter the adsorption and activation modes of CO_2_. The reaction energy of COOH^−^ formation from ^•^CO_2_^−^ – the rate‐limiting step – can be lowered on the Br sites. Based on these advantages, Cs_3_Bi_2_Br_9_ exhibited an improved CO yield of 134.76 µmol g^−1^ with a 98.7% selectivity. This study showed that the halogen sites, which are generally produced in the solution‐processed MHPs, could serve as a highly powerful tool to enhance photocatalytic performance by optimizing the electronic structure and modulating CO_2_ adsorption.

On the other hand, despite the defects on the surface of MHPs have been shown to boost the photocatalytic CO_2_ reduction, the stability of the photocatalysts was not discussed in the above studies. Note that it has been reported that the defects act as the recombination center for charge carriers, which will lead to the deactivation of MHPs during the photocatalytic process.^[^ ^7^, ^8^ ^]^ To examine the effect, Chen et al. investigated the electronic properties of Cs_2_AgBiBr_6_ with and without Br‐vacancy.^[^ ^116^ ^]^ The calculated density of states (DOS) plots showed that the presence of Br vacancy causes the movement of the fermi level from VBM to near the CBM (Figure 13F), where the peak resulted from defective states and separated from the relatively delocalized electrons in the CB, and the exceeding electrons caused by Br vacancy are localized. Most importantly, the introduced extra defective electronic states originating from the Br vacancies were very close to the CBM, which were defined as a shallow energy level and could not accelerate the deactivation of the Cs_2_AgBiBr_6_. Similarly, calculation results also demonstrated that CO_2_ molecules can be chemically and preferably adsorbed on Br‐vacancy Cs_2_AgBiBr_6_, and Br‐vacancy decreases the potential determining step energy.

Note that besides the chemical methods, light irradiation is also another feasible approach for the defect formation in MHPs, and this is experimentally confirmed by Wang et al. in Cs_3_Sb_2_I_9_ material.^[^ ^11c^ ^]^ In this study, in‐situ EPR data indicated that the I defect active sites were generated on the Cs_3_Sb_2_I_9_ surface by the thermal excitation (Figure 13G). Using this material for photocatalytic CO_2_ reduction without sacrificial agents or cocatalysts, the CH_4_ and CO production rate of 95.7 µmol g^−1^ h^−1^ was achieved through the photothermal synergistic effect, which is 87‐ and 5.2‐fold higher than that of pure photocatalysis and pure thermal catalysis, respectively (Figure 13H). Detailed characterization results showed that the defects active sites on Cs_3_Sb_2_I_9_ facilitate the adsorption and activation of CO_2_ (Figure 13I), meanwhile the plentiful electrons excited by light provided the CO_2_ reduction capacity, and thus the synergistically improved the photoactivity of CO_2_ reduction.

To sum up, the above studies have shown that the presence of certain defects in MHP materials is indeed beneficial for the photocatalytic CO_2_ reaction, and engineering defects of MHPs could be a promising strategy to improve the photocatalytic performance of MHPs. Nevertheless, currently the defects are mainly focused on halogen (i.e., Cl, Br, and I) vacancies, while the A site (such as Cs) and B site (Ag, Sb, etc.) have not been investigated. Thus, there is still plenty of room to be explored. On the other hand, although the defect can be confirmed by combining a set of advanced characterizations (such as EPR and XPS) and the defect formation can be achieved through different approaches, the exact concentration of defects cannot be identified, and the process of engineering defects is still uncontrollable. As discussed above, the presence of these intrinsic point defects is highly dependent on their formation energy and the growth conditions. The frequently adopted solution process for the preparation of MHP is the lack of sophisticated control on both the nucleation and growth processes for MHP materials. Therefore, finer elaboration of synthesis methods with more deliberation might be helpful to attain the MHP‐based photocatalysts with more docile defects. These issues need to be taken into account in further research in order to achieve the precise manipulation of the MHP photocatalysis.

#### 4.2.2 H_2_ Generation

H_2_ is one of the most promising clean and sustainable energy carrier to replace traditional fossil fuels.^[^ ^119^ ^]^ Photocatalytic H_2_ evolution, generated from the splitting of water, hydrohalic acid, or alcohols, is an attractive renewable technology. In 2016, Nam et al. first demonstrated the photocatalytic H_2_ generation over MHPs,^[^ ^120^ ^]^ in which the saturated HI acid aqueous solution was adopted to address the instability issue of MAPbI_3_. Since then, the application of MHPs in H_2_ evolution has been further exploited through coupling MHPs with other semiconductors or electron cocatalysts. Until now, great efforts have been made to improve the photocatalytic performance of MHPs in photocatalytic H_2_ evolution.^[^ ^121^ ^]^ However, the photocatalytic activity over MHPs is still relatively low and thus more efficient approaches need to be developed.

**Figure 14:**
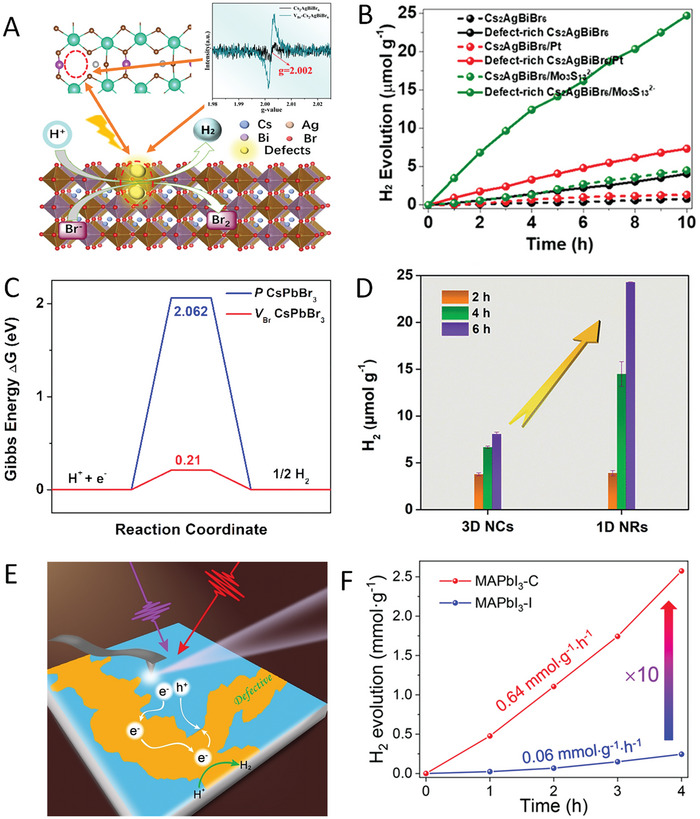
A) Scheme of the photocatalytic H_2_ evolution over the Br defect‐rich Cs_2_AgBiBr_6_ photocatalyst. B) H_2_ generation rate over Br‐rich Cs_2_AgBiBr_6_ and reference samples. Reproduced with permission.^[^ ^11e^ ^]^ Copyright 2021, American Chemical Society. C) The Gibbs free energy plots of CsPbBr_3_ with and without Br vacancies for photocatalytic H_2_ generation. D) The H_2_ production rate over pristine CsPbBr_3_ (3D NCs) and Br‐rich CsPbBr_3_ (1D NRs). Reproduced with permission.^[^ ^122^ ^]^ Copyright 2023, Royal Society of Chemistry. E) Schematic illustration of the charge dynamics in MAPbI_3_ with and without the continuation of defective areas for solar‐driven H_2_ generation. F) H_2_ generation rate over MAPbI_3_‐I and MAPbI_3_‐C photocatalysts. Reproduced with permission.^[^ ^11b^ ^]^ Copyright 2023, Wiley‐VCH.

Previous reports have suggested that the construction of defects was favorable for photocatalytic H_2_ generation because the surface defects can decrease the number of coordinated active sites and promote the surface charge separation.^[^ ^38^ ^]^ This was also confirmed by He et al. in Cs_2_AgBiBr_6_ MHPs (**Figure** **14**A).^[^ ^11e^ ^]^ They showed that the absorption edge of the Br vacancy in Cs_2_AgBiBr_6_ exhibits a red shift due to the change of local atomic arrangement and electronic structure, and the narrowed bandgap was also confirmed by DFT. Meanwhile, experimental characterizations showed that the defective Cs_2_AgBiBr_6_ promotes electron transfer. As a result, the photocatalytic H_2_ evolution over defect‐rich Cs_2_AgBiBr_6_/Mo_3_S_13_^2−^ reached 24.7 µmol g^−1^ within 10 h of visible light irradiation, which was 5.5 times enhancement compared to pristine Cs_2_AgBiBr_6_/Mo_3_S_13_^2−^ (Figure 14B). Besides, this photocatalyst presented excellent stability with no obvious performance decrease after 80 h of recycling tests. Following this work, Zhang and coworkers also engineered Br vacancies on Cs_2_AgBiBr_6_ for H_2_ evolution.^[^ ^11d^ ^]^ The DFT results showed the enriched Br vacancies on Cs_2_AgBiBr_6_ (VBr‐Cs_2_AgBiBr_6_) surfaces introduced effective active sites for H_2_ evolution. When coupling VBr‐Cs_2_AgBiBr_6_ with WO_3_, a further improved photocatalytic H_2_ evolution rate of 364.89 µmol g^−1^ h^−1^ was achieved, with long‐term stability of 12 h continuous reaction.

Besides Cs_2_AgBiBr_6_, the beneficial effect of defects in photocatalytic H_2_ generation was also demonstrated in 1 D CsPbBr_3_ by Guo et al.,^[^ ^122^ ^]^ where the existence of Br vacancies in CsPbBr_3_ (V_Br_‐CsPbBr_3_) was experimentally confirmed. Through DFT calculations, a reduced Gibbs energy was observed with Br vacancies (Figure 14C). Also, the spectroscopic results showed the facilitated photogenerated charge carrier separation and transfer in CsPbBr_3_ with fewer Br vacancies. Note that this work also pointed out a few Br vacancies are favorable for proton reduction, while large amounts of Br vacancies have a negative effect on photogenerated electron‐hole separation. Taken together, the optimal 1D nanorods V_Br_‐CsPbBr_3_ displayed a fivefold improvement for photocatalytic H_2_ evolution (Figure 14D). Although the above studies reported an enhanced H_2_ generation in defective MHP photocatalysts, the MHPs used in these systems are all‐inorganic MHP materials. Does this similar effect exist in organic‐inorganic hybrid MHPs? To answer this question, Yao et al. deliberately synthesized a MAPbI_3_ (MAPbI_3_‐C) photocatalyst featuring a unique continuation of I defective areas (Figure 14E).^[^ ^11b^ ^]^ Through a set of advanced techniques, including microscopy‐based infrared, space‐resolved PL, and femtosecond time‐resolved transient absorption spectroscopies, they showed that the continuation of defective areas not only retards the electron‐trapping process but also prolongs the lifetime of photogenerated electrons in MAPbI_3_. As a result, MAPbI_3_‐C showed an H_2_ production rate of 0.64 mmol g^−1^ h^−1^, one order of magnitude greater than that of conventional MAPbI_3_ (Figure 14F).

In summary, these studies showed the engineered defects on MHPs offer a powerful route to optimize the electronic structure and optoelectronic properties of MHPs which ultimately contribute to the photocatalytic H_2_ generation. Note that defects have dual functions, i.e., excess defects unavoidable act as the detrimental recombination center for the photocatalytic performance.^[^ ^38^ ^]^ However, the threshold of defect density that promotes the H_2_ generation rate was still not studied precisely. Therefore, it is anticipated that the defect amount/concentration can be controlled by manipulating the synthesis process, collaborating with the theoretical calculation, to maximize the positive effect of defects. On the other hand, how the Br vacancies in Cs_2_AgBiBr_6_ and CsPbBr_3_, and the continuously distributed defects in MAPbI_3_ contributed to the H_2_ evolution, i.e., whether the defect favors the H adsorption and H_2_ desorption, or it alters the reaction energy barrier, or the other combined effects, is still unclear and should be further investigated to better understand the mechanisms. Considering only a few publications indicated that the engineered defects on the surface of MHPs are beneficial for photocatalytic H_2_ production so far, more investigations still need to be done to extend this strategy for the boosted photocatalytic H_2_ evolution.

#### 4.2.3 Pollutant Degradation

Apart from the photocatalytic CO_2_ reduction and H_2_ evolution for the generation of solar fuels, MHPs have been shown to be powerful in photocatalytic pollutant degradation,^[^ ^123^ ^]^ in which the organic pollutants will be degraded to biodegradable compounds or less toxic molecules, and ultimately mineralize them into CO_2_ and H_2_O.

However, most MHPs are not stable under practical reaction conditions, therefore the design of more stable MHP photocatalysts is of great importance.^[^ ^7b^ ^]^ Recently, Pb‐free double‐perovskites by replacing Pb^2+^ with Sn^2+^, Ge^2+^ and Bi^3+^ have been reported.^[^ ^124^ ^]^ Among these alternatives, Cs_2_AgBiBr_6_ has triggered appealing interest due to its good stability. Nevertheless, the relatively low photoredox ability restricts its further development in photocatalytic pollution degradation. To address this issue, tuning the electronic structures of MHPs via the introduction of defects would be useful.

**Figure 15:**
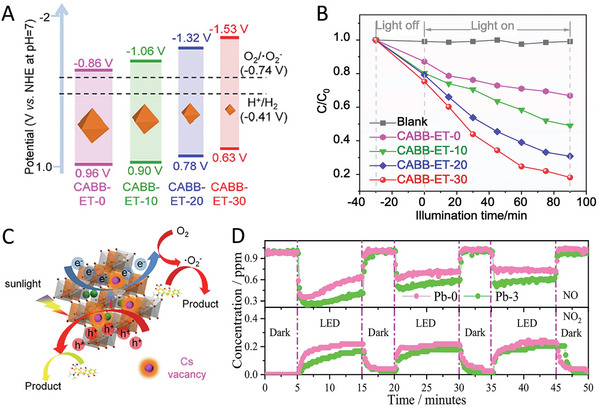
A) Schematic illustrating band structures of Cs_2_AgBiBr_6_ samples, where CABB‐ET‐0, 10, 20, and 30 represent adding of Ethylenediaminetetraacetic acid disodium salt (ET) with different evaporation time (0, 10, 20, and 30 min). B) Photodegradation performance of tetracycline over various Cs_2_AgBiBr_6_. C) The proposed mechanism for photodegradation of the tetracycline over CABB‐ET samples. Reproduced with permission.^[^ ^11f^ ^]^ Copyright 2022, Elsevier. D) Photocatalytic NO oxidation over Cs_3_Bi_2−_ *_x_* Pb*_x_* Br_9−_ *_x_* photocatalysts. Reproduced with permission.^[^ ^100^ ^]^ Copyright 2023, The Authors, published by Springer.

In recent work, Zhang et al. reported that the generation of Cs vacancies in Cs_2_AgBiBr_6_ promotes the formation of surface defects to control its electronic structure.^[^ ^11f^ ^]^ The optimized Cs_2_AgBiBr_6_ has the strongest reduction capacity with the conduction band potential of −1.53 V (vs NHE) to date (**Figure** **15**A), which can greatly promote the production of superoxide radicals (^•^O_2_^–^), improving the photocatalytic efficiency of the Cs_2_AgBiBr_6_. Besides, experimental results also showed an efficient charge separation in defective Cs_2_AgBiBr_6_. Under solar light irradiation for 90 min, the photodegradation efficiency of tetracycline for the as‐obtained Cs_2_AgBiBr_6_ with a surface defect up to 81.8% (Figure 15B). Thus, combined with the effective charge dynamics and the formation of ^•^O_2_^–^, the enhanced photocatalytic performance was illustrated in Figure 15C.

As confirmed by this work, the photoredox performance of MHPs depends not only on their light‐harvesting properties but also on the surface chemical environment during their synthesis. To further reveal the role of the defect on the photocatalytic pollution degradation, Gualdron‐Reyes et al. studied the influence of the surface chemical environment of CsPbBr_3−_ *_x_* I*_x_*.^[^ ^106^ ^]^ X‐ray photoelectron spectroscopy and surface photovoltage analyses showed that the CsPbBr_3−_ *_x_* I*_x_* with iodide vacancies are the main surface defects that facilitate the formation of ^•^O_2_^–^, and lead to a better photocatalytic activity for the oxidation of β‐naphthol. Beyond these reports, Chen et al. studied the dopant‐induced Br vacancy in Cs_3_Bi_2_Br_9_ for photocatalytic NO oxidation,^[^ ^100^ ^]^ in which partially replacing Bi^3+^ with Pb^2+^ yielded the Br vacancy in the Cs_3_Bi_2−_ *_x_* Pb*_x_* Br_9−_ *_x_* (0 ≤ x ≤ 0.0789) samples. Impressively, the formed Br vacancy favors NO adsorption and activation and visible‐light harvesting. Also, an increased ionic selectivity was found in the oxidized NO being absorbed on Br vacancy sites. As a result, a significantly increased NO oxidation efficiency (80%) was achieved under LED (λ≥400 nm) irradiation over defect‐containing Cs_3_Bi_2−_ *_x_* Pb*_x_* Br_9−_ *_x_* (*x* = 0.0443) photocatalyst (Figure 15D).

To summarize, defect engineering on MHPs has been demonstrated to tune the optical properties, charge dynamics, and photoredox potentials, and those are the key factors affecting the pollutant degradation performance of MHP‐based photocatalysts. It is true that the oxidation ability and ^•^O_2_^–^ radicals are highly associated with pollutant degradation, while the efficient adsorption of reactants and desorption of products on the surface of MHPs are also vital to achieving higher photodegradation performance. Nevertheless, in the above studies, the multiple factors were not explored in depth. This may be due to a few works on the defects engineering of MHPs for pollutant degradation so the symmetrical investigation has not yet been conducted. Thus, further research is anticipated to give an in‐depth insight into the effects from various aspects. Besides, both cation (such as Cs) and anion (such as I) vacancies are claimed to promote the production of superoxide ^•^O_2_^–^ and thus improve the photocatalytic performance of pollutant degradation, while the difference between these two kinds of vacancies on capture ^•^O_2_^–^ as well as their oxidation capabilities were not revealed. It is important to distinguish the exact roles of cation and anion‐induced defects to fully utilize the benefits of defects for photocatalytic pollutant degradation.

#### 4.2.4 Organic Synthesis

Besides the aforementioned applications, MHPs have also been exploited for photocatalytic organic synthesis, such as the polymerization of TerEDOT, α‐alkylation reaction of aldehydes, and C‐C bond formation via C‐H activation, N‐heterocyclization, C‐O cross‐coupling, and Suzuki coupling.^[^ ^109^, ^125^ ^]^ Although various types of reactions are demonstrated to perform well, no direct report on defects that help to improve the photocatalytic performance of organic synthesis so far. So, the raised question is: do the defects can also be applied to boost the organic synthesis reactions? To reveal the nature of the reaction, some recent works that investigated the reactive sites, activation and conversion effect, and surface reaction pathway associated with the MHPs may elucidate this perplexity, with two representative examples present here.

**Figure 16:**
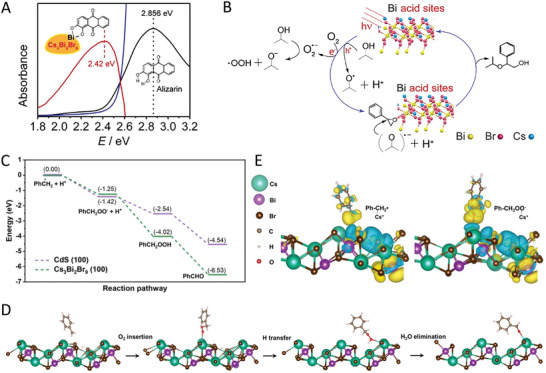
A) UV‐Vis spectra of Cs_3_Bi_2_Br_9_ materials adsorbed with (red) and without alizarin (blue), and pure alizarin (black). B) Illustrating photocatalytic epoxide alcoholysis over Cs_3_Bi_2_Br_9_ sample with Lewis acid sites. Reproduced with permission.^[^ ^126^ ^]^ Copyright 2019, Wiley‐VCH. C) Reaction energy profile of toluene oxidation over Cs_3_Bi_2_Br_9_ and CdS. (D‐E) Schematic illustration showing D) different key intermediates that adsorbed on Cs_3_Bi_2_Br_9_ surface and E) charge density difference of adsorbed intermediates. Reproduced with permission.^[^ ^127^ ^]^ Copyright 2022, Elsevier.

For instance, Dai et al. investigated the effect of active sites on the photocatalytic performance of ring‐opening reactions of using Cs_3_Bi_2_Br_9_ photocatalyst.^[^ ^126^ ^]^ Under visible light irradiation in air at room temperature, Cs_3_Bi_2_Br_9_ displayed high activity (1333 µmol g^−1^ h^−1^) and selectivity (86%) to produce 2‐isopropoxy‐2‐phenylethanol from styrene oxide and isopropanol, much higher than that of Bi‐free MHPs. Surface acidity characterization (**Figure** **16**A) and control experiments showed that the high photocatalytic activity was highly associated with the exposed Bi‐based Lewis‐acid sites on the surface of Cs_3_Bi_2_Br_9_, which play a key role in activating the epoxides (Figure 16B). This work confirmed that the active sites, which normally can be manipulated through defect engineering, have a significant impact on photocatalysis.

Following this concept, a recent work from Zou's group further studied the activation effect and surface reaction pathway of Cs_3_Bi_2_Br_9_ sites in Cs_3_Bi_2_Br_9_/CdS heterojunction for C(sp^3^)‐H bond activation.^[^ ^127^ ^]^ Focusing on the photocatalytic toluene oxidation, DFT calculations showed that Cs_3_Bi_2_Br_9_ with exposed Cs and Br sites is more thermodynamically favorable for the conversion of key intermediates (PhCH_2_⋅ and PhCH_2_OO^−^) compared to CdS (Figure 16C). Specifically, the benzyl radical (PhCH_2_⋅) and H^+^ are adsorbed on Cs_3_Bi_2_Br_9_ via C‐Cs and H‐Br bond, respectively. ^•^O_2_^−^ and PhCH_2_⋅ produce PhCH_2_OO^−^ intermediate via a single O‐Cs bond. PhCH_2_OO^−^ and H^+^ further form PhCH_2_OOH with an adsorption structure through another O‐Cs bond, followed by the elimination of H_2_O from PhCH_2_OOH to generate the desired PhCHO product (Figure 16D). These results collaborated with the experimental observations. This work demonstrated the synergistic effects of Cs/Br sites for the surface activation and reaction steps (Figure 16E), which are considered as the functional components for the photocatalytic reactions and should be given more attention.

Summarizing, the defects in MHPs have been shown to potentially improve the photocatalytic activity of organic transformation. The defects can offer more active sites, promote the reaction pathway, and facilitate the adsorption/desorption processes. However, the direct design of the defects in MHPs for photocatalytic organic synthesis has not been reported, and thus more experimental results are needed to confirm and elaborate the impact of the defects. Besides, employing advanced techniques to demonstrate the existing defects in MHPs is anticipated in further research. Moreover, how the defects in different photocatalytic organic synthesis systems contribute to the photoredox processes and the essential mechanisms require deeper investigation, which could offer a guideline for moving the defect‐mediated MHPs in organic synthesis forward.

Put together, the above subsections discussed the specific examples in which the defects in MHPs were utilized to enhance the photocatalytic performance of MHP‐based photocatalysts, with detailed information summarized in **Table** **1**. Regarding the synthesis approaches of defects in MHP photocatalysts, wet chemical methods including solution process, solvothermal, ligand exchange, and antisolvent recrystallization are widely adopted due to the solution‐processing characteristics of MHPs. Some other strategies like grinding and post‐treatment using light irradiation can also be feasible. This is slightly different from traditional photocatalysts, and interested readers are recommended to delve into the referenced literature.^[^ ^10a,b^ ^]^ In terms of the characterization techniques of defects, spectroscopic methodology and microscopic techniques are the two main tools.^[^ ^10b^ ^]^ Various spectroscopic methods, including XPS, electron paramagnetic resonance (EPR), and UV‐Vis diffuse reflectance spectroscopy, have been employed to identify defects in MHP photocatalysts.^[^ ^10c^ ^]^ Each of these techniques provides unique structural information about the photocatalysts, often complementing each other in the process of defect identification. For instance, XPS is widely used to study the surface atomic components and the chemical states of defects. EPR is capable of detecting unpaired electrons of photocatalysts. Given the close relationship between defects and atoms and excess electrons, EPR serves as an ideal technique for identifying defects in semiconductors.^[^ ^10b,c^ ^]^ Furthermore, UV‐Vis can determine how defects alter the light absorption behavior of photocatalysts. Additionally, microscopic techniques such as TEM, scanning tunneling microscopy (STM) and atomic force microscopy (AFM) provide a direct way to observe the atomic structures of defects.^[^ ^10a^ ^]^ STM, in particular, can reveal changes in the local surface potential of defective photocatalysts, making it a promising technique for visualizing defects in photocatalysts. Also, AFM is an effective tool for directly scanning the surface of defective photocatalysts at the nanoscale level.^[^ ^10a,b^ ^]^ Note that although the defect engineering of MHPs contributes to the photocatalysis, the photoactivity in the above four types of reactions (CO_2_ reduction, H_2_ generation, pollutant degradation, and organic synthesis) still needs to be improved. This can be achieved by developing new synthesis approaches and precisely controlling the defect type and its concentration. Nevertheless, compared with the generous MHP compounds, the ones currently in use are very limited (mainly focus on Cs_2_AgBiBr_6_ and Cs_3_Bi_2_Br_9_). Therefore, exploring more MHP materials is another feasible route to move the MHP defect‐based photocatalysis forward.

**Table 1:** Summary of the recent advancements in engineering defects of MHPs for photocatalytic applications.

MHP photocatalyst	Defect type	Synthesis method	Identify method	Main functions of the defects	Photocatalytic application	Reaction condition	Photocatalytic activity	Measured time	Ref.
Cs_2_NaBiCl_6_	Cl vacancies	Grinding	XPS and EPR	Enhancing CO_2_ adsorption and reducing free energy of intermediate species	CO_2_ reduction	Gas (CO_2_+H_2_O), 300 W Xe lamp	CO: 30.22 µmol g^−1^ h^−1^ CH_4_: 1.12 µmol g^−1^ h^−1^	50 h	[11a]
Cs_3_Sb_2_Br_9_	Br vacancies	Hot injection	XPS	Facilitating charge separation	CO_2_ reduction	CO_2_+H_2_O, AM 1.5 G (300 W Xe lamp, 100 mW cm^−2^)	CO: 510 µmol g^−1^	4 h	[117]
Cs_2_AgBiBr_6_	Br vacancies	Epitaxial growth	XPS and EPR	Facilitating charge separation	CO_2_ reduction	CO_2_+H_2_O, AM 1.5 G (300 W Xe lamp, 150 mW cm^−2^)	CH_4_: 22.6 µmol g^−1^ h^−1^	16 h	[11g]
Cs_3_Bi_2_Br_9_	Br vacancies	Antisolvent recrystallization	XPS	Enhancing CO_2_ adsorption and activation	CO_2_ reduction	CO_2_+H_2_O, AM 1.5 G (300 W Xe lamp)	CO: 134.76 µmol g^−1^	20 h	[118]
Cs_3_Sb_2_I_9_	I vacancies	Modified solution process	EPR	Facilitating charge separation	CO2 reduction	CO2+H2O, Xe lamp, 200 mW cm^−2^, 235 oC	CO+CH_4_: 95.7 µmol g^−1^ h^−1^	12 h	[11c]
Cs_2_AgBiBr_6_	Br vacancies	Light treatment	XPS and TEM	Enhancing light absorption and facilitating charge separation	H_2_ generation	HBr aqueous, visible light (λ > 420 nm, 332.5 mW cm^–2^)	H_2_: 24.7 µmol g^−1^	10 h	[11e]
Cs_2_AgBiBr_6_	Br vacancies	Solvothermal	TEM and EPR	Facilitating charge separation and reaction kinetics	H_2_ generation	HBr/H_3_PO_2_ aqueous, visible light (λ > 420 nm, 100 mW cm^–2^)	H_2_: 364.89 µmol g^−1^ h^−1^	12 h	[11d]
CsPbBr_3_	Br vacancies	Ligand exchange	XPS	Facilitating charge separation and proton reduction	H_2_ generation	HI/H_3_PO_2_ aqueous, visible light (λ > 400 nm, 300 W Xe lamp)	H_2_: ≈240 µmol g^−1^	20 h	[122]
MAPbI_3_	Continuous MA defective areas	Solution growth	XPS	Facilitating charge separation	H_2_ generation	HI/H_3_PO_2_ aqueous, visible light (λ > 420 nm, 300 W Xe lamp)	H_2_: 0.64 mmol g^−1^ h^−1^	28 h	[11b]
Cs_2_AgBiBr_6_	Cs vacancies	Solution process	XPS	Promoting superoxide radical generation	Pollutant degradation	AM 1.5 G (100 mW cm^–2^, 300 W Xe lamp)	Tetracycline photodegradation (81.8%, 90 min)	480 min	[11f]
CsPbBr_3−_ *_x_* I*_x_*	I vacancies	Anion exchange	XPS	Facilitating the formation of superoxide radical	Pollutant degradation	Visible light (150 W lamp, 60 Mw cm^–2^)	β‐naphthol photodegradation (61%, 90 min)	90 min	[106]
Cs_3_Bi_2−_ *_x_* Pb*_x_* Br_9−_ *_x_*	Br vacancies	Solution process	EPR	Facilitating NO adsorption and activation and enhancing light absorption	Pollutant degradation	LED lamp (λ≥ 400 nm)	NO oxidation efficiency (80%, 50 min)	50 min	[100]
Cs_3_Bi_2_Br_9_	Bi acid sites	Solution process	UV‐Vis	Enhancing the epoxide activation	Organic synthesis	Visible light (λ > 420 nm, 300 W Xe lamp)	2‐isopropoxy‐2‐phenylethanol: 1333 µmol h^−1^ g^−1^	18 h	[126]
Cs_3_Bi_2_Br_9_	Br active sites	Solution process	XPS	Enhancing toluene adsorption and activation	Organic synthesis	Visible light (λ > 420 nm, Xe lamp)	Benzaldehyde: 6.79 mmol g^−1^ h^−1^	12 h	[127]

## 5 Conclusions and Outlook

In summary, MHP materials have emerged as promising photocatalysts due to their excellent optoelectronic properties and low‐cost solution processing. However, the effect of the defects of MHPs on the photocatalytic performance remains ambiguous. In this review, we have articulated the physics origin of defects in MHPs and appraised the role of the defects in MHP‐based photocatalysis. More specifically, the recent advances in the utilization of defects to boost the MHP‐based photocatalytic reactions, including CO_2_ reduction, H_2_ generation, pollutant degradation, and organic synthesis, are recapitulated and discussed. Despite the outstanding achievements, taking advantage of defects of MHPs in photocatalytic applications is still in its infancy and faces some challenges. More efforts are still needed to be made to design, fabricate, modify, characterize, and utilize defects to fully explore their potentials in photocatalysis. Based on the assessment of recent progress in the field of defect‐based MHP photocatalysis, some directions for future research are proposed as follows:

First, synthesis approaches. Due to the easy control of compositions, MHPs with a great number of varieties can be synthesized, and the potential to modify these MHPs through defect engineering extrapolates this number literally to infinity. However, for the current research, the method for the fabrication of defects is limited and even not given in some studies. On the other hand, because of the water‐sensitive properties of MHPs and the phase transformation in high temperatures, the defects in most MHPs cannot be achieved through the traditional approaches as compared to the perovskite oxides counterparts, such as hydrothermal, sol‐gel, impregnation, incipient wetness, molten salt, pechini, and thermal calcination.^[^ ^38^ ^]^ Therefore, the development of more useful techniques that lead to the equilibration and achievement of steady‐state performance is vital. Defects from solid solubility, doping, and light soaking may be feasible strategies for designing the defects in MHPs.

Second, characterization techniques. Identifying the defects is vital to understanding the influence of defects on photocatalytic reaction processes and offers a guideline to rational design certain defects for boosting photocatalytic performance. So far, some characterization techniques such as XPS, EPR, photoelectron spectroscopy, and STEM, have been developed to examine the defects. However, these techniques can generally convince the existence of defects, while the type, concentration, and corresponding local atomic environment cannot be fully identified. Those are highly associated with massive external conditions and intrinsic properties of MHP materials, thus developing more advanced characterization technologies or following the approaches that used in MHP‐based photovoltaics, is essential for accurately designing defective sites in MHPs, which would help to reduce the negative effects in photocatalytic processes, such as defects serving as recombination centers for photogenerated electron‐hole pairs. For instance, drive‐level capacitance profiling (DLCP) method was adopted to provide a spatial and energetic distribution of the trap states as well as the densities of the free carriers and traps of the semiconductors, which has been reported in Si,^[^ ^128^ ^]^ Cu_2_ZnSnSe_4_,^[^ ^129^ ^]^ and MHPs.^[^ ^35^ ^]^ It is reasonable to deduce that the mature characterization techniques in other fields are possible to serve as the boost in the defect property analysis of MHP‐based photocatalysts if proper adaptation could be made.

Thirdly, the optimization of functions. Surface defects in MHPs were demonstrated to play vital roles in photocatalytic applications. By introducing different surface defects, the crucial steps of the photocatalytic processes (light absorption, separation and transport of charge carriers, and surface redox reactions) can be significantly optimized. Especially, halogen vacancies (V_X_: X = I, Br, and Cl) have been shown to enhance the photocatalytic performance. Even with these successes in MHPs, some difficulties and challenges still need to be resolved. For example, both cation (Cs, Bi) and anion (Br, I) have been declared to tune electron structure and thus contributing to the improved photocatalytic performance. However, the difference between these two kinds of defects is not clarified, and their functions still need further investigation. On the other hand, under certain conditions, the defects can act as recombination centers for electron‐hole pairs during photocatalytic processes. Hence, precisely understanding the roles of defects in different MHPs is extremely important, namely, control and maximization of appropriate types and concentrations of defects as active sites.

Fourthly, the reliability of defects. Functions and structural stabilization of the MHPs with surfaces and local defects should be unraveled simultaneously, and the development of techniques for defect engineering requires to be decoupled. This depends greatly on the potential to exploit existing technologies for synthesis, characterization, and simulation and to develop new ones. In this sense, the unambiguous characterization of defects and elaborate clarification of their functions in photocatalytic processes are necessary but remain challenging. Although theoretical calculation is a powerful approach to the study of defects, the simulated model cannot reflect the authentic structure of the catalyst. Therefore, advanced techniques, especially in situ observation, should be developed to evaluate the active sites and catalytic mechanism during the photocatalytic process, which is of great importance for designing defective structures and even for accelerating the development of materials science.

Fifthly, the stability issue. Some studies showed the defects in MHPs will not compromise the stability of the MHP photocatalysts, this is to some certain degree contradictory to previously reported in MHP‐based solar cells. Thus, the relationship between the defects (surface or bulk defects, the type of defects) and the structural stability requires further in‐depth insights. With unremitting endeavor regarding these challenges, researchers will make increasingly more convincing and important contributions in defect engineered MHP materials to catalysis science – through a combination of experimental dataG, advanced analytical techniques, and simulations to reveal defective MHPs and the defect equilibria in photocatalysis – whereby we can look forward to greatly improved photocatalytic performance for practical applications.

Finally, the mechanism subject. Regarding the mechanism issue, two aspects require in‐depth analysis. One is how the defects formed and what is the driving force. For example, even though using the same synthesis method, some researchers claimed the formation of Br vacancies in MHPs while others proposed there are Cs vacancies.^[^ ^11^, ^100^ ^]^ The underlying mechanism is still unclear and needs to be investigated carefully. Another one is about the main function(s) of the defects, it varies in different studies. Taking the Cs_2_AgBiBr_6_ as an example, when applied to CO_2_ photoreduction, it can be proposed to facilitate charge separation or enhance CO_2_ adsorption and activation.^[^ ^11e,g^ ^]^ This inconsistency could be alleviated by combining a set of experimental characterizations and DFT calculations. With these issues addressed, it is anticipated that more excellent MHP photocatalysts can be developed by the means of defect engineering.

## Conflict of Interest

The authors declare no conflict of interest.
